# Supplementary material for: Immediate hydroxylation of arenes to phenols via V-containing all-silica ZSM-22 zeolite triggered non-radical mechanism
Source: Nat Commun. 2018 Jul 26;9:2931. doi: 10.1038/s41467-018-05351-w (PMC6062531; doi:10.1038/s41467-018-05351-w)
Supplement: Supplementary file 1 — Supplementary Information [file 41467_2018_5351_MOESM1_ESM.pdf]

Supplementary Information

Immediate hydroxylation of arenes to phenols via V-containing all-silica ZSM-22 zeolite triggered non-radical mechanism

Zhou et al.

Supplementary methods

Synthesis of V_2O_5@Si-ZSM-22

V_2O_5-impregnated ZSM-22 sample with similar V content to VSZ-5 was prepared through wet-impregnation method. Si-ZSM-22 (1 g) was mixed with 10 mL aqueous NH_4VO_3 solution and stirred at room temperature for 10 h. After that, the mixture was evaporated at 100 °C to remove the water. The solid was calcined at 550 °C for 5 h to give the final product V_2O_5@Si-ZSM-22.

Synthesis of V-AlSi-ZSM-22

V- and Al-containing ZSM-22 with similar V content to VSZ-5 was synthesized with the same procedure as VSZ-5 except that aluminum sulfate was added before [BMIm]Br. The obtained sample was named as V-AlSi-ZSM-22.

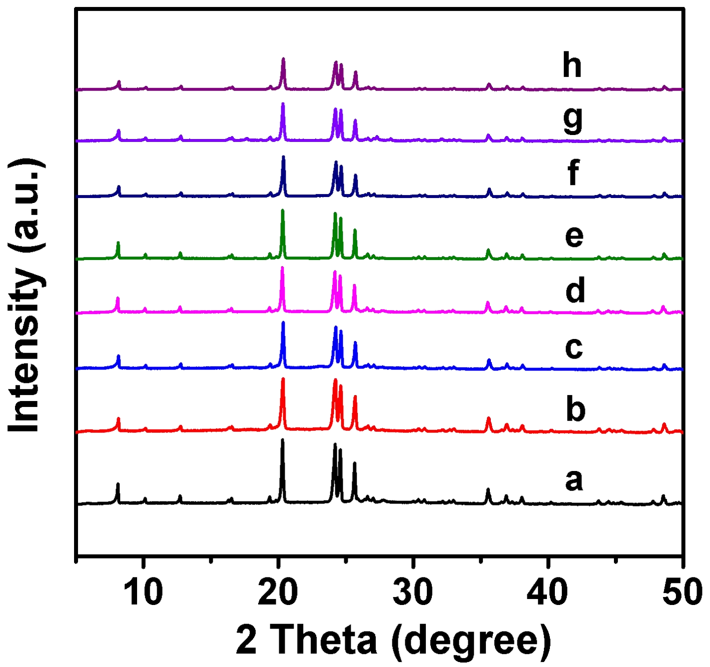

Supplementary Figure 1 | XRD patterns. (a) Si-ZSM-22, (b) VSZ-1, (c) VSZ-3, (d) VSZ-5, (e) VSZ-10, (f) VSZ-15, (g) VSZ-20 and (h) VSZ-25.

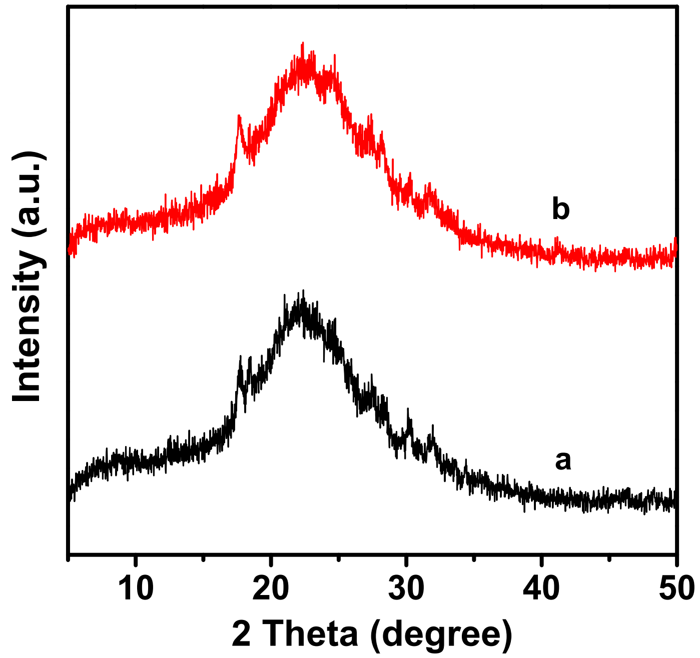

Supplementary Figure 2 | XRD patterns. The samples were synthesized with the initial composition of (a) 0.30 and (b) 0.35 NH_4VO_3: SiO_2: 0.35 [BMIm]Br: 0.2 Na_2O: 40 H_2O.

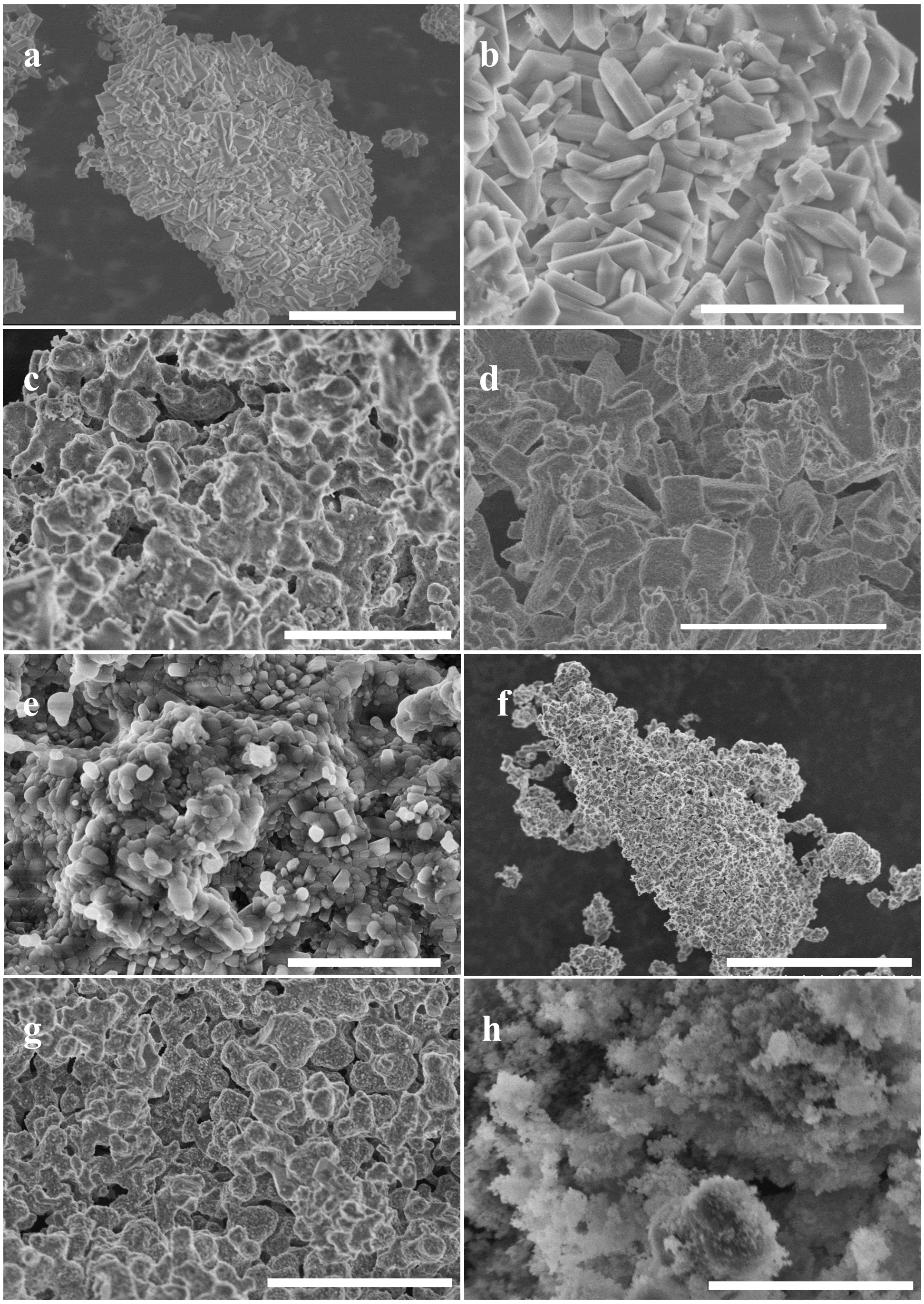

Supplementary Figure 3 | SEM images. (a) Si-ZSM-22, (b) VSZ-1, (c) VSZ-3, (d) VSZ-5, (e) VSZ-10, (f) VSZ-15, (g) VSZ-20 and (h) VSZ-25. Scale bars, 20 μm (a), 5 μm (b), 10 μm (c), 10 μm (d), 2 μm (e), 50 μm (f), 5 μm (g), 5 μm (h).

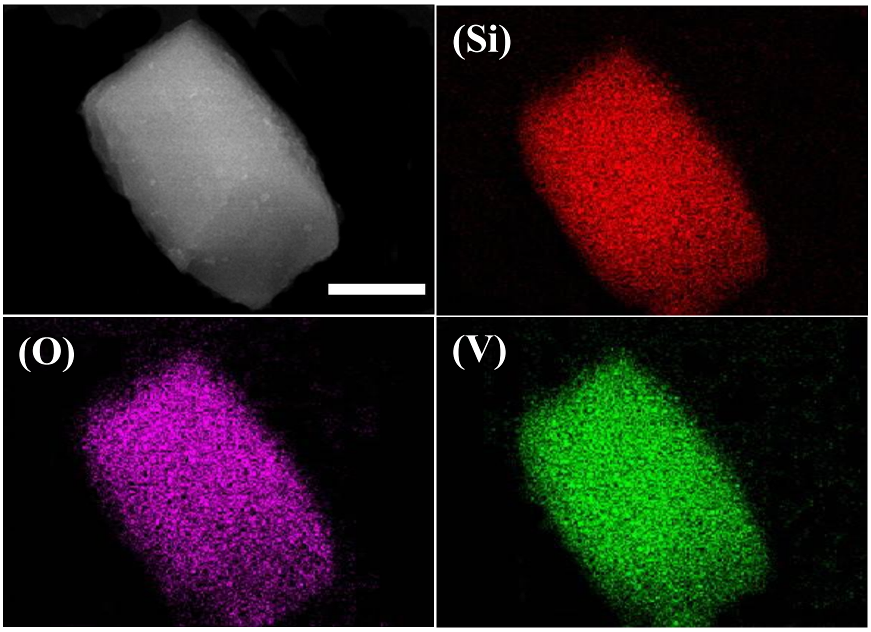

Supplementary Figure 4 | Elemental mapping analysis. SEM image and energy-dispersive X-ray spectrometry (EDS) elemental mapping images (Si, O and V element) of VSZ-5. Scale bar, 5 μm.

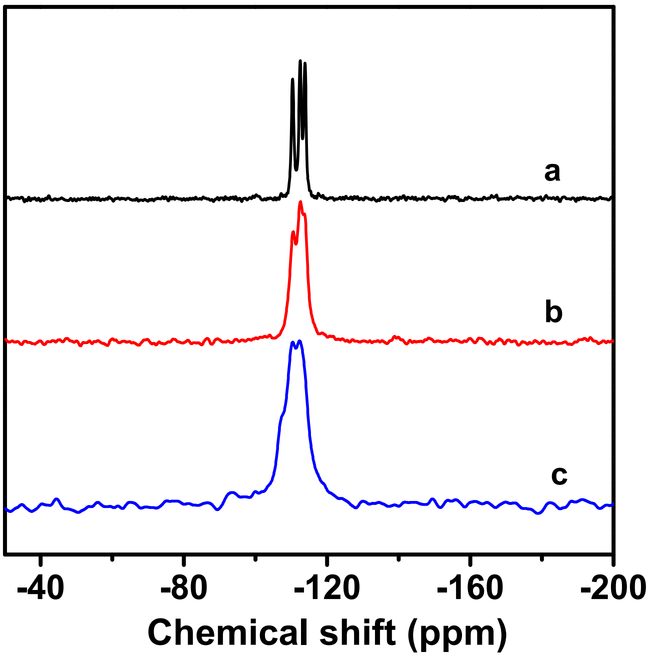

Supplementary Figure 5 | ^{29}Si MAS NMR spectra. (a) VSZ-5, (b) VSZ-5(m) and (c) VSZ-5(r1).

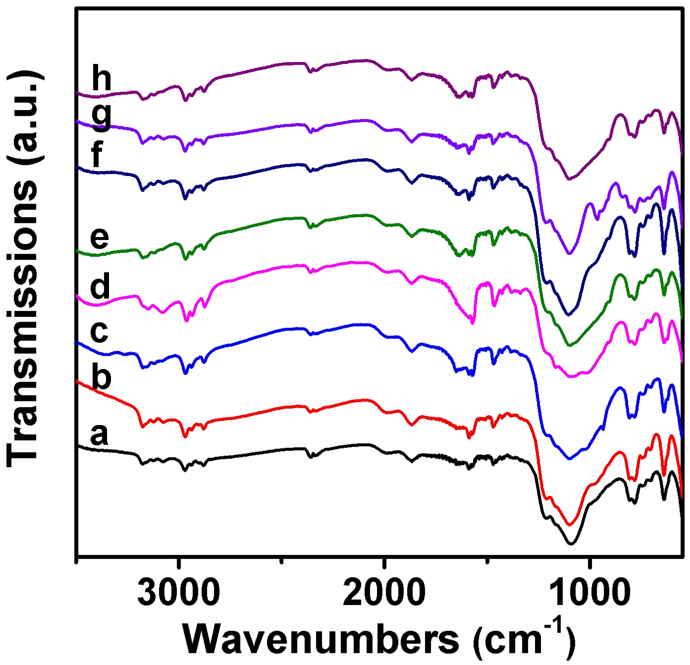

Supplementary Figure 6 | FTIR spectra of as-synthesized samples. (a) Si-ZSM-22, (b) VSZ-1, (c) VSZ-3, (d) VSZ-5, (e) VSZ-10, (f) VSZ-15, (g) VSZ-20 and (h) VSZ-25.

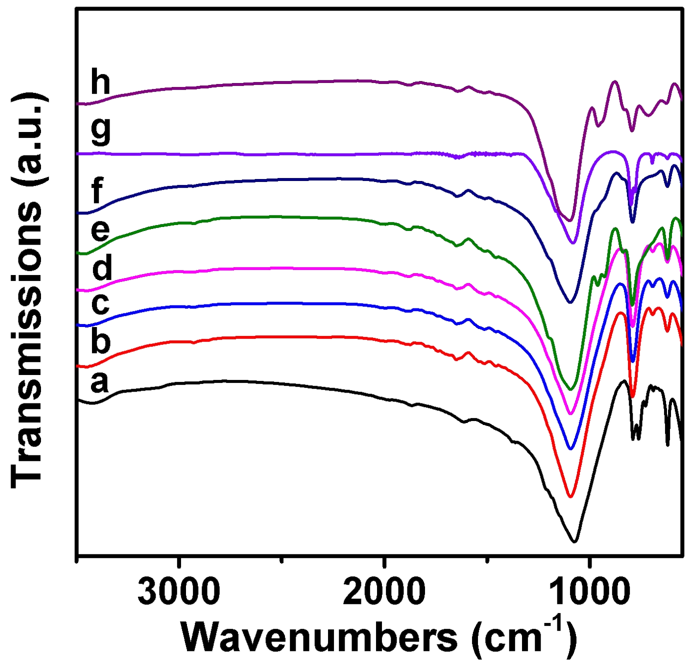

Supplementary Figure 7 | FTIR spectra. (a) Si-ZSM-22, (b) VSZ-1, (c) VSZ-3, (d) VSZ-5, (e) VSZ-10, (f) VSZ-15, (g) VSZ-20 and (h) VSZ-25.

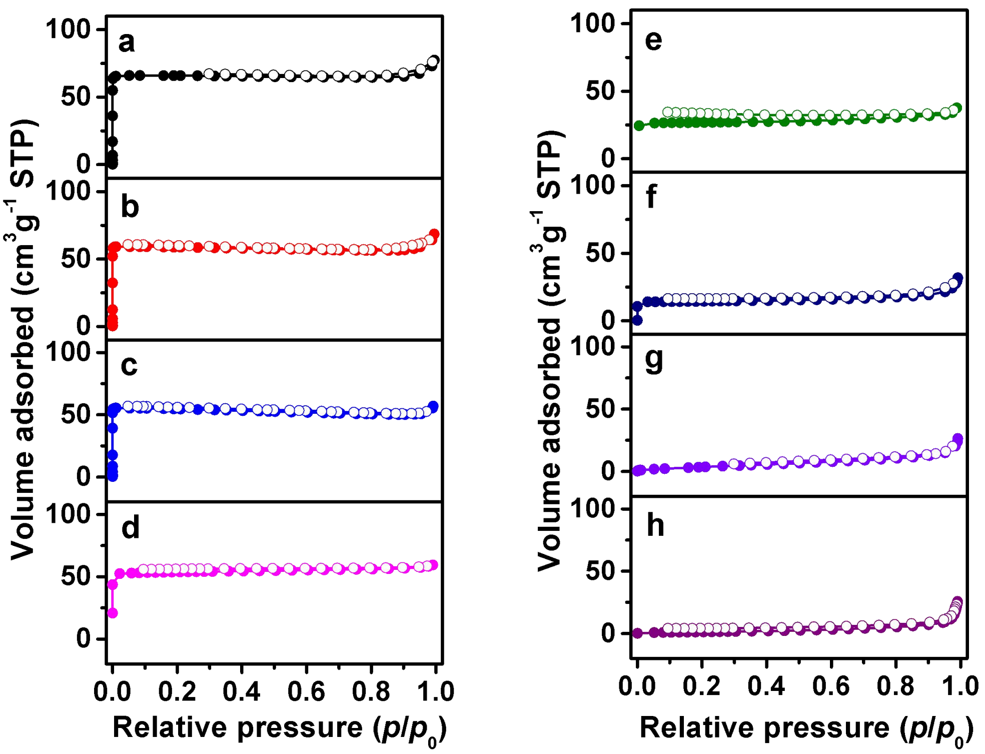

Supplementary Figure 8 | N_2 sorption isotherms. (a) Si-ZSM-22, (b) VSZ-1, (c) VSZ-3, (d) VSZ-5, (e) VSZ-10, (f) VSZ-15, (g) VSZ-20 and (h) VSZ-25.

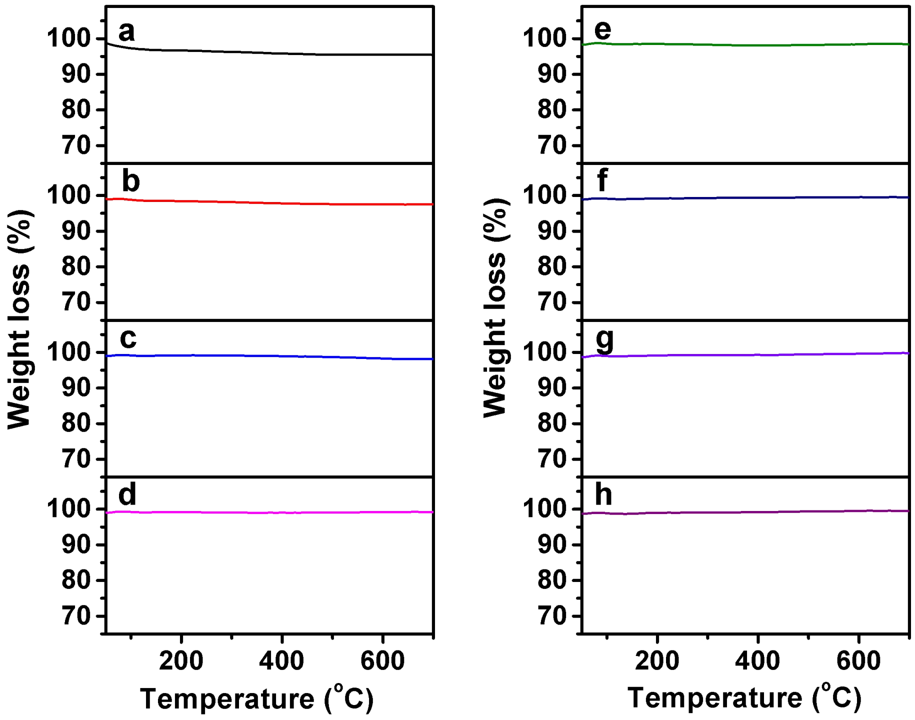

Supplementary Figure 9 | TG curves. (a) Si-ZSM-22, (b) VSZ-1, (c) VSZ-3, (d) VSZ-5, (e) VSZ-10, (f) VSZ-15, (g) VSZ-20 and (h) VSZ-25.

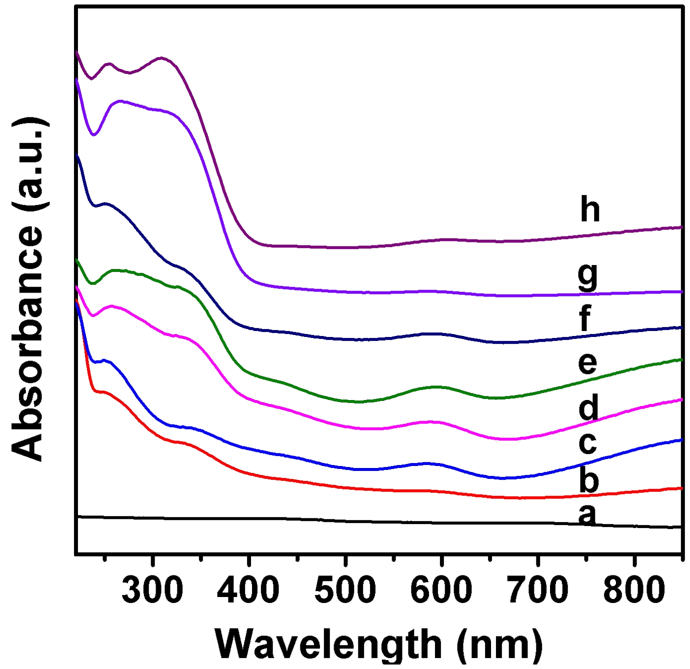

Supplementary Figure 10 | UV spectra of as-synthesized samples. (a) Si-ZSM-22, (b) VSZ-1, (c) VSZ-3, (d) VSZ-5, (e) VSZ-10, (f) VSZ-15, (g) VSZ-20 and (h) VSZ-25.

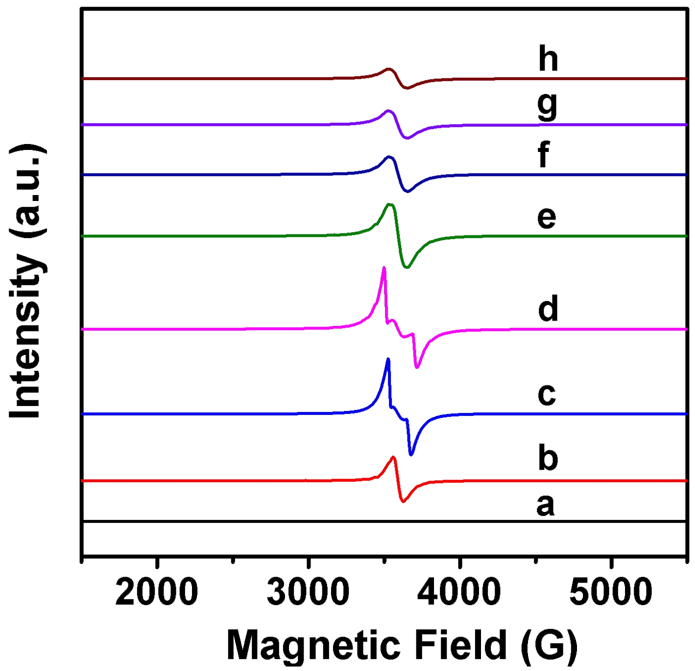

Supplementary Figure 11 | ESR spectra of as-synthesized samples. (a) Si-ZSM-22, (b) VSZ-1, (c) VSZ-3, (d) VSZ-5, (e) VSZ-10, (f) VSZ-15, (g) VSZ-20 and (h) VSZ-25.

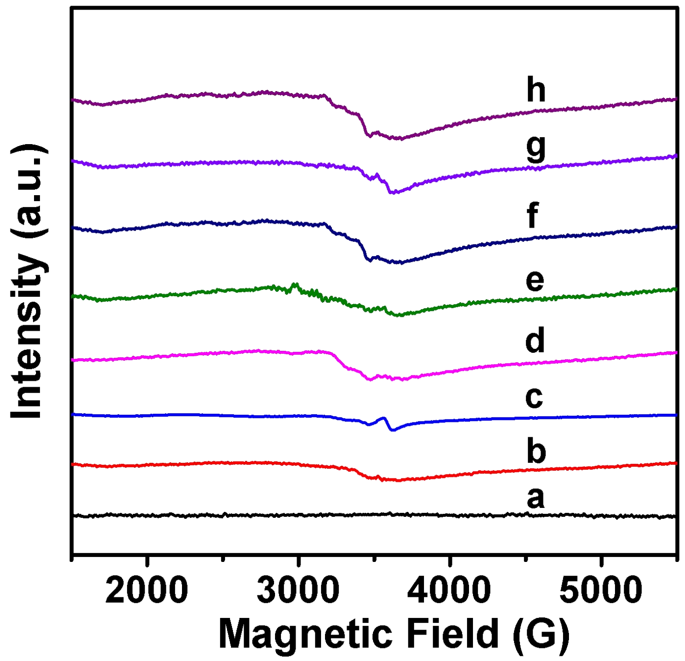

Supplementary Figure 12 | ESR spectra. (a) Si-ZSM-22, (b) VSZ-1, (c) VSZ-3, (d) VSZ-5, (e) VSZ-10, (f) VSZ-15, (g) VSZ-20 and (h) VSZ-25.

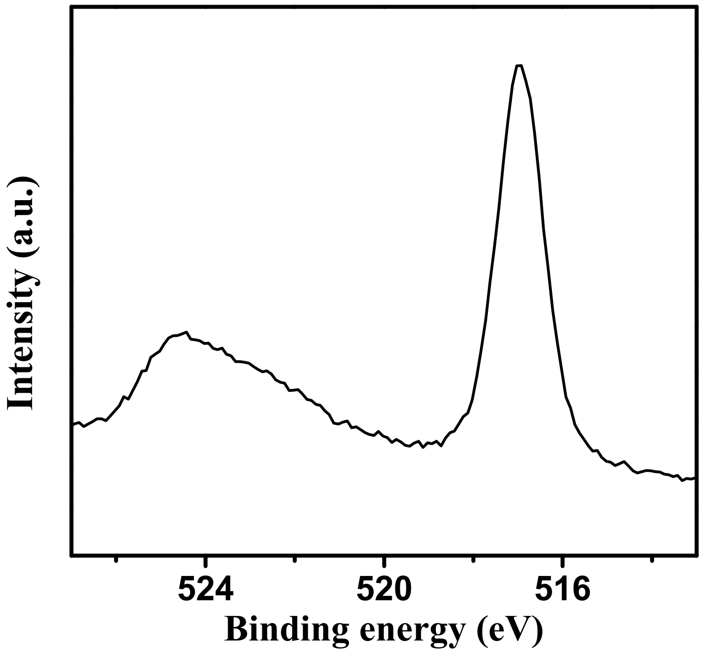

Supplementary Figure 13 | XPS analysis. V2p XPS spectrum of VSZ-5.

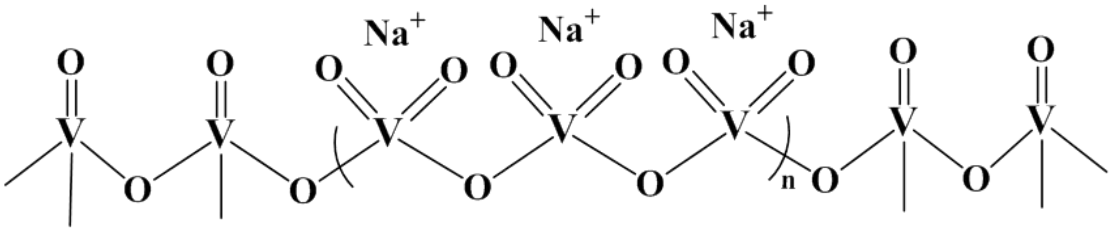

Supplementary Figure 14 | Proposed structure. $V_2O_7^{4-}$ species serve as the bridge to link the $(VO_3)_n^{n-}$ species and silica framework.

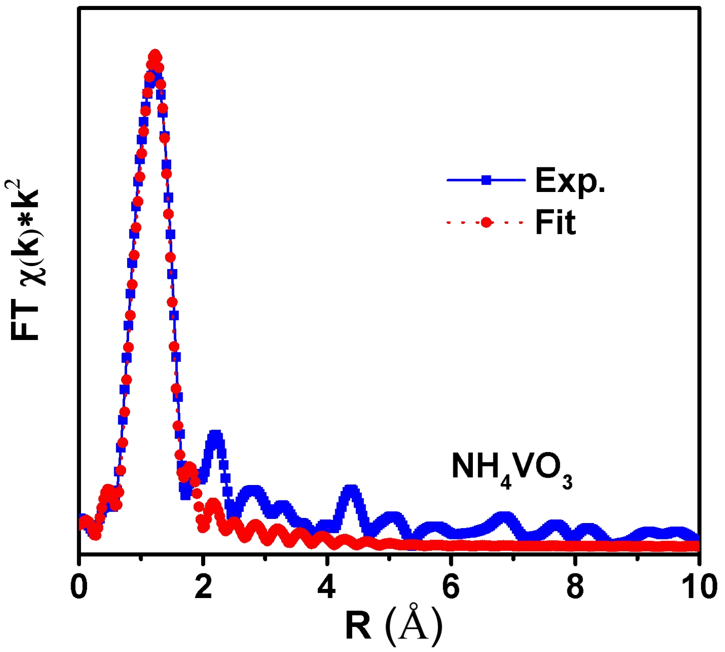

Supplementary Figure 15 | FT-EXAFS curves. The experimental data and the fit for NH_4VO_3.

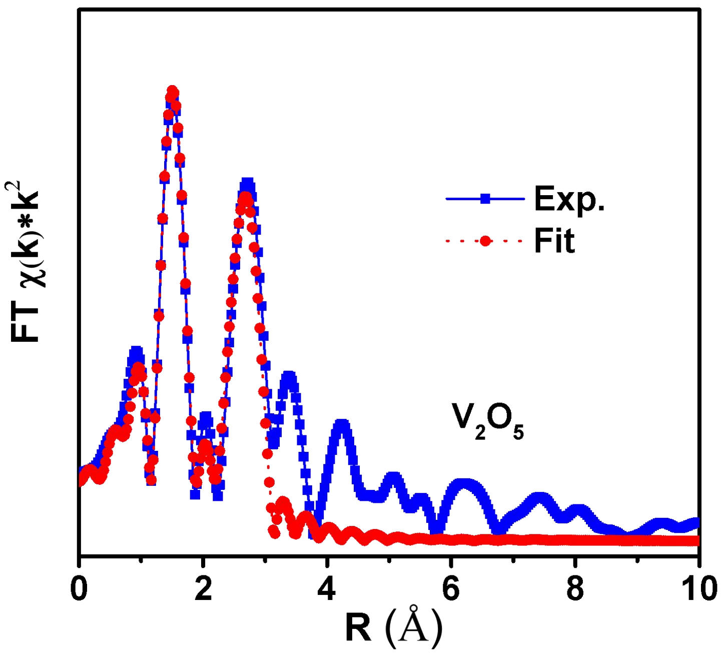

Supplementary Figure 16 | FT-EXAFS curves. The experimental data and the fit for V_2O_5.

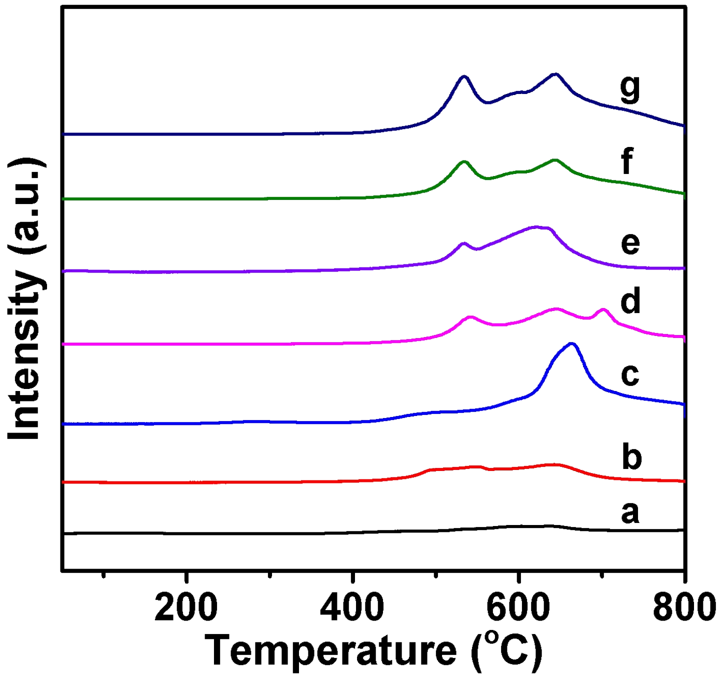

Supplementary Figure 17 | H_2-TPR profiles. (a) VSZ-1, (b) VSZ-3, (c) VSZ-5, (d) VSZ-10, (e) VSZ-15, (f) VSZ-20 and (g) VSZ-25.

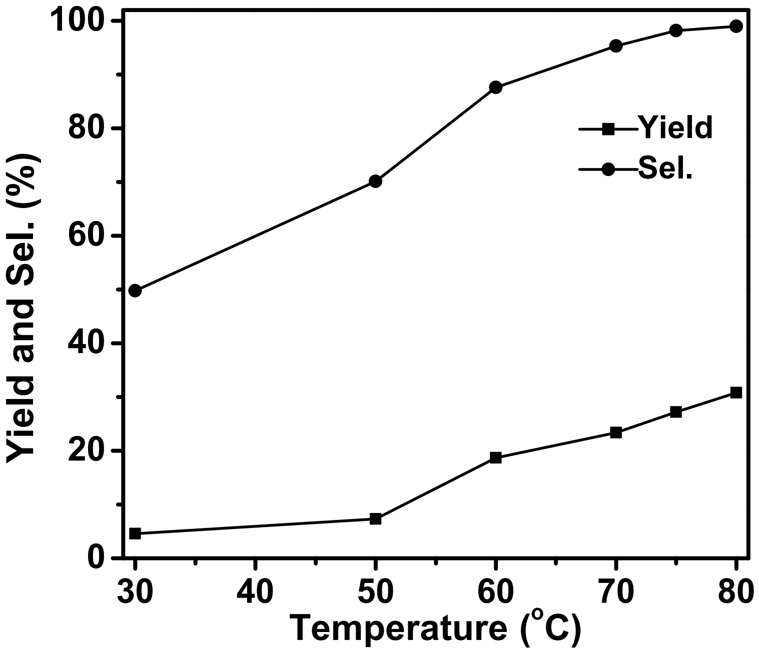

Supplementary Figure 18. | Yield and selectivity as a function of reaction temperature in VSZ-5 catalyzed hydroxylation of benzene with H_2O_2. Reaction condition: benzene (5 mmol), aqueous H_2O_2 (30%, 5 mmol), VSZ-5 (0.1 g), H_2SO_4 (0.15 g), CH_3CN (14 mL), 80 °C, 30 s. Yield (%): [phenol (mol)/initial benzene (mol)]×100. Selectivity (%): [phenol (mol)/converted benzene (mol)]×100.

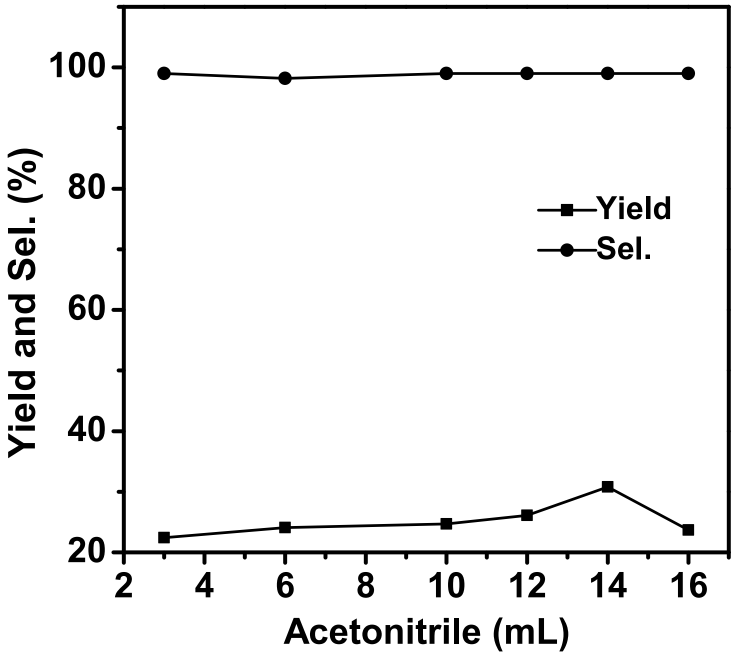

Supplementary Figure 19 | Yield and selectivity as a function of CH_3CN amount in VSZ-5 catalyzed hydroxylation of benzene with H_2O_2. Reaction condition: benzene (5 mmol), aqueous H_2O_2 (30%, 5 mmol), VSZ-5 (0.1 g), H_2SO_4 (0.15 g), CH_3CN, 80 °C, 30 s. Yield (%): [phenol (mol)/initial benzene (mol)]×100. Selectivity (%): [phenol (mol)/converted benzene (mol)]×100.

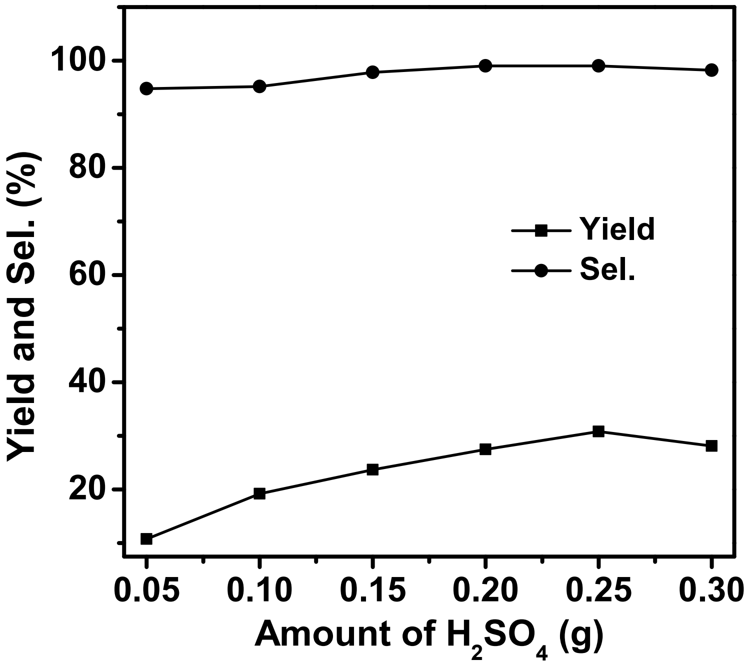

Supplementary Figure 20 | Yield and selectivity as a function of H_2SO_4 amount in VSZ-5 catalyzed hydroxylation of benzene with H_2O_2. Reaction condition: benzene (5 mmol), aqueous H_2O_2 (30%, 5 mmol), VSZ-5 (0.1 g), H_2SO_4, CH_3CN (14 mL), 80 °C, 30 s. Yield (%): [phenol (mol)/initial benzene (mol)]×100. Selectivity (%): [phenol (mol)/converted benzene (mol)]×100.

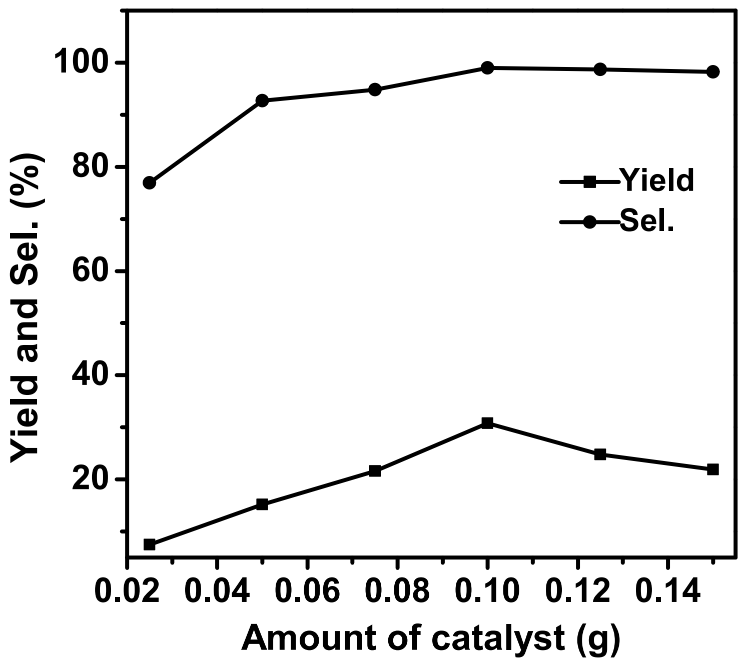

Supplementary Figure 21 | Yield and selectivity as a function of catalyst dosage in VSZ-5 catalyzed hydroxylation of benzene with H_2O_2. Reaction condition: benzene (5 mmol), aqueous H_2O_2 (30%, 5 mmol), VSZ-5, H_2SO_4 (0.15 g), CH_3CN (14 mL), 80 °C, 30 s. Yield (%): [phenol (mol)/initial benzene (mol)]×100. Selectivity (%): [phenol (mol)/converted benzene (mol)]×100.

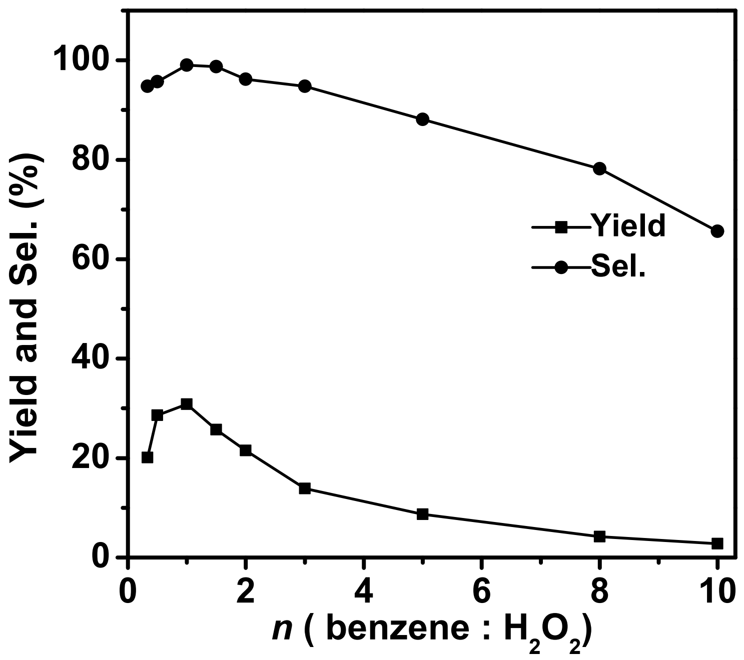

Supplementary Figure 22 | Yield and selectivity as a function of benzene/H_2O_2 ratio in VSZ-5 catalyzed hydroxylation of benzene with H_2O_2. Reaction condition: benzene (5 mmol), aqueous H_2O_2 (30%), VSZ-5 (0.1 g), H_2SO_4 (0.15 g), CH_3CN (14 mL), 80 °C, 30 s. Yield (%): [phenol (mol)/initial benzene (mol)]×100. Selectivity (%): [phenol (mol)/converted benzene (mol)]×100.

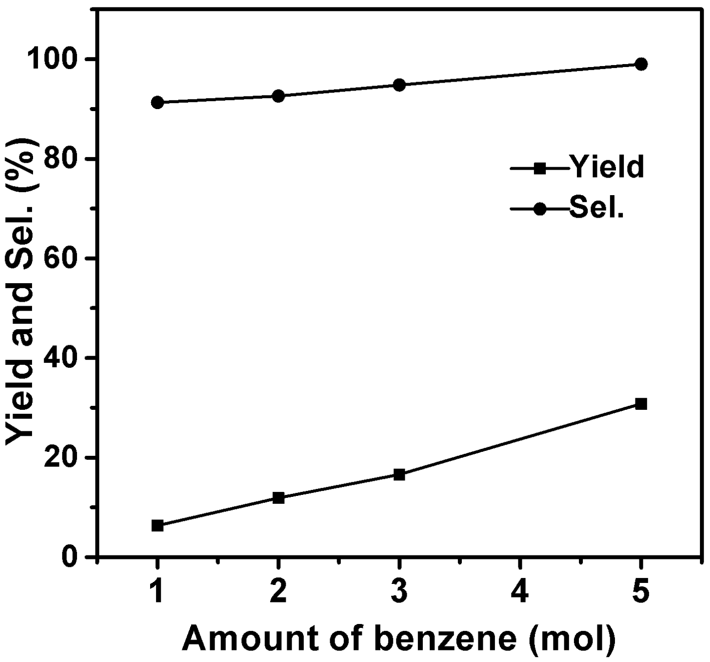

Supplementary Figure 23 | Yield and selectivity as a function of amount of benzene in VSZ-5 catalyzed benzene hydroxylation with H_2O_2. Reaction condition: benzene (5 mmol), aqueous H_2O_2 (30%, 5 mmol), VSZ-5, H_2SO_4 (0.15 g), CH_3CN (14 mL), 80 °C, 30 s. Yield (%): [phenol (mol)/initial benzene (mol)]×100. Selectivity (%): [phenol (mol)/converted benzene (mol)]×100.

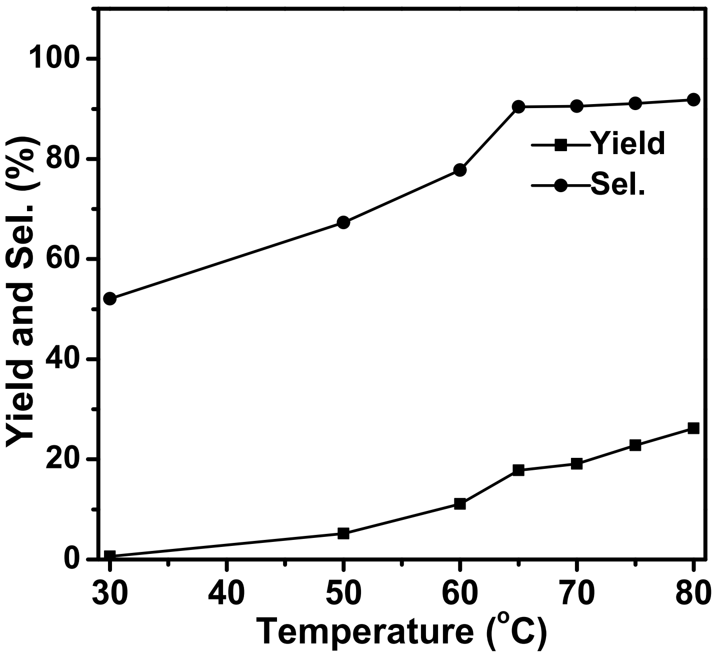

Supplementary Figure 24 | Yield and selectivity as a function of reaction temperature in VSZ-5 catalyzed toluene hydroxylation with H_2O_2. Reaction condition: toluene (5 mmol), aqueous H_2O_2 (30%, 5 mmol), VSZ-5 (0.1 g), H_2SO_4 (0.15 g), CH_3CN (12 mL), 30 s. Yield (%): [cresols (mol)/initial toluene (mol)]×100. Selectivity (%): [cresols (mol)/converted toluene (mol)]×100.

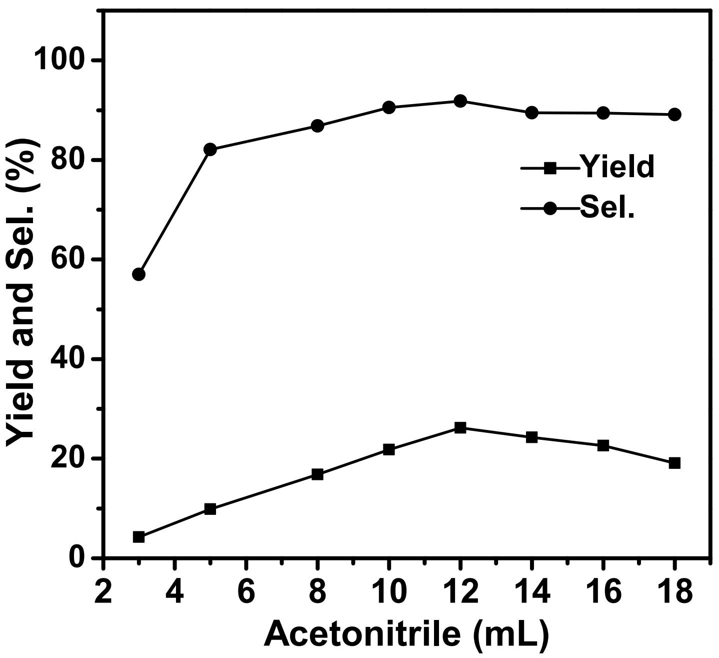

Supplementary Figure 25 | Yield and selectivity as a function of CH_3CN amount in VSZ-5 catalyzed toluene hydroxylation with H_2O_2. Reaction condition: toluene (5 mmol), aqueous H_2O_2 (30%, 5 mmol), VSZ-5 (0.1 g), H_2SO_4 (0.15 g), CH_3CN, 80 °C, 30 s. Yield (%): [cresols (mol)/initial toluene (mol)]×100. Selectivity (%): [cresols (mol)/converted toluene (mol)]×100.

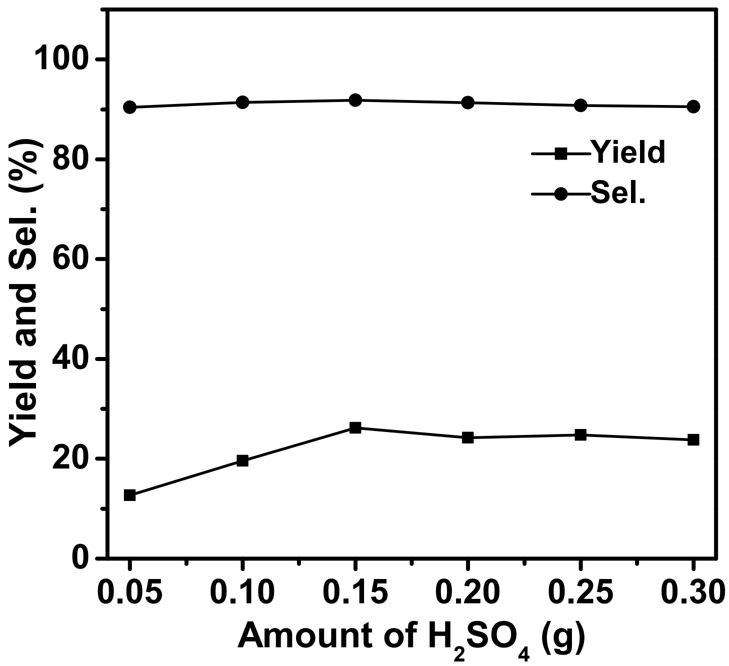

Supplementary Figure 26 | Yield and selectivity as a function of H_2SO_4 amount in VSZ-5 catalyzed toluene hydroxylation with H_2O_2. Reaction condition: toluene (5 mmol), aqueous H_2O_2 (30%, 5 mmol), VSZ-5 (0.1 g), H_2SO_4, CH_3CN (12 mL), 80 °C, 30 s. Yield (%): [cresols (mol)/initial toluene (mol)]×100. Selectivity (%): [cresols (mol)/converted toluene (mol)]×100.

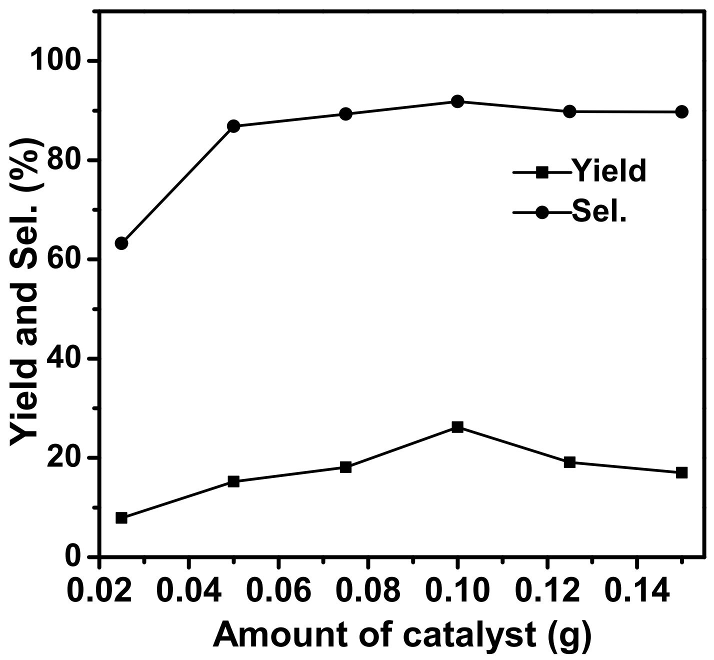

Supplementary Figure 27 | Yield and selectivity as a function of catalyst dosage in VSZ-5 catalyzed toluene hydroxylation with H_2O_2. Reaction condition: toluene (5 mmol), aqueous H_2O_2 (30%, 5 mmol), VSZ-5, H_2SO_4 (0.15 g), CH_3CN (12 mL), 80 °C, 30 s. Yield (%): [cresols (mol)/initial toluene (mol)]×100. Selectivity (%): [cresols (mol)/converted toluene (mol)]×100.

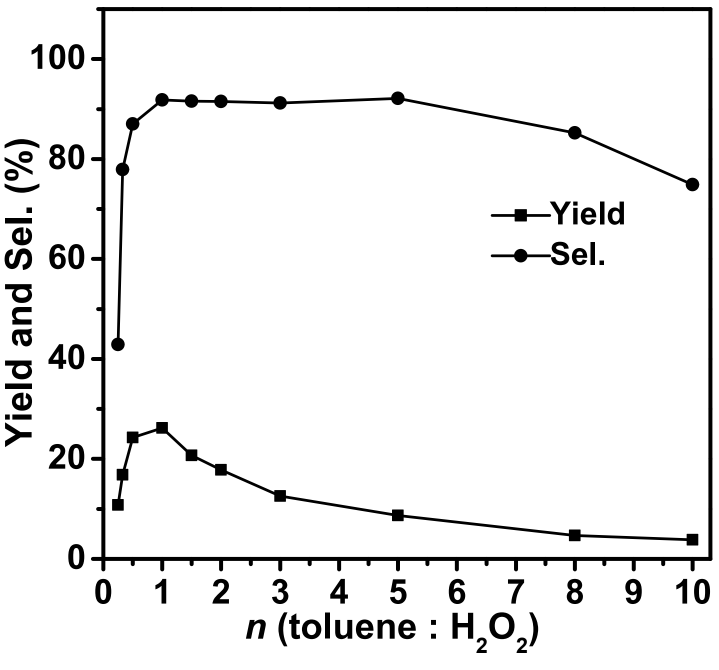

Supplementary Figure 28 | Yield and selectivity as a function of toluene/H_2O_2 ratio in VSZ-5 catalyzed toluene hydroxylation with H_2O_2. Reaction condition: toluene (5 mmol), aqueous H_2O_2 (30%), VSZ-5 (0.1 g), H_2SO_4 (0.15 g), CH_3CN (12 mL), 80 °C, 30 s. Yield (%): [cresols (mol)/initial toluene (mol)]×100. Selectivity (%): [cresols (mol)/converted toluene (mol)]×100.

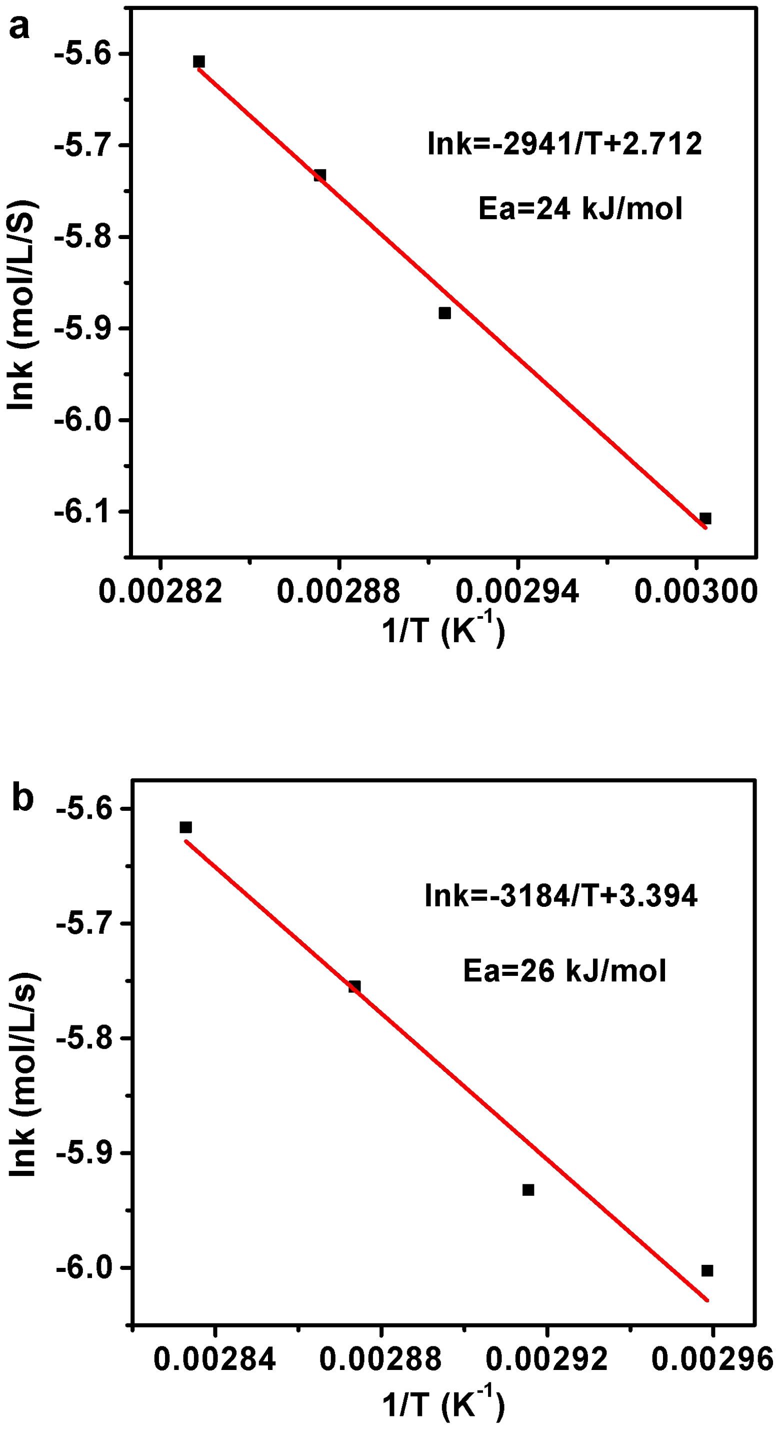

Supplementary Figure 29 | Calculation of the activation energy barrier. Linear plot of lnk vs. 1/T in VSZ-5 catalyzed hydroxylation of (a) benzene and (b) toluene. The lnk and 1/T was calculated from Supplementary Figs. 18 and 24.

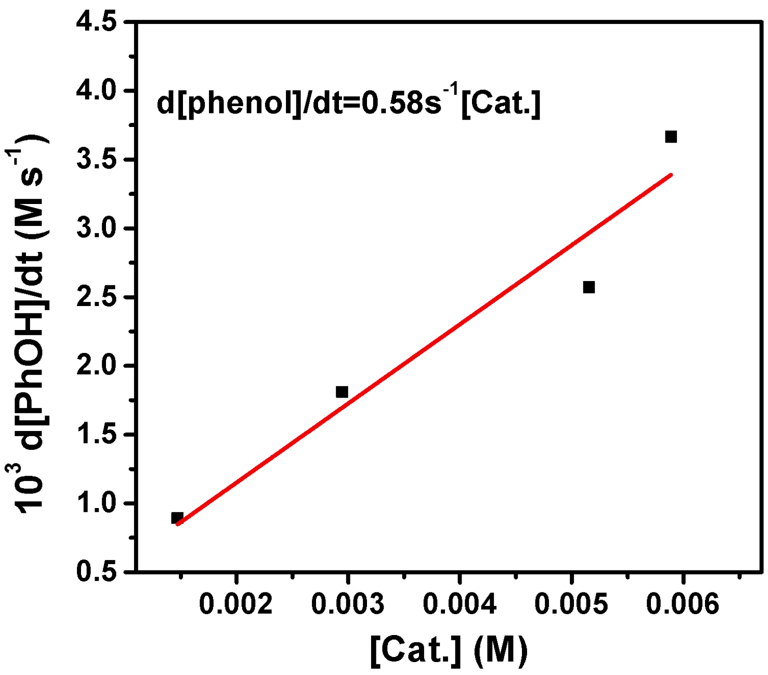

Supplementary Figure 30 | Linear plot of the reaction rate (d[PhOH]/dt) vs. centration of catalyst VSZ-5. Reaction conditions: benzene (5 mmol), aqueous H_2O_2 (30%, 5 mmol), VSZ-5 (0.025~0.1 g), H_2SO_4 (0.15 g), CH_3CN (14 mL), 80 °C, 30 s. d[PhOH]/dt was calculated from [PhOH]/t at 30 s (Supplementary Fig. 21) by assuming that the kinetic curve belongs to a first-order equation. The concentration of catalyst VSZ-5 was calculated based on the molar amount of V sites: [molar amount of V in VSZ-5]/[volume of the reaction solution].

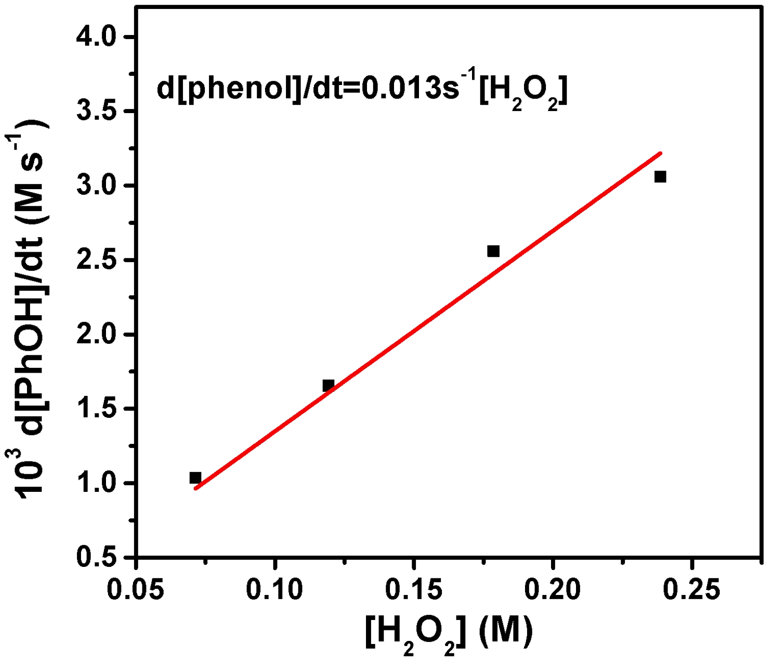

Supplementary Figure 31 | Linear plot of the reaction rate (d[PhOH]/dt) vs. centration of H_2O_2. Reaction conditions: benzene (5 mmol), aqueous H_2O_2 (30%, 1 ~ 3.3 mmol), VSZ-5 (0.1 g), H_2SO_4 (0.15 g), CH_3CN (14 mL), 80 °C, 30 s. d[PhOH]/dt was calculated from [PhOH]/t at 30 s (Supplementary Fig. 22) by assuming that the kinetic curve belongs to a first-order equation.

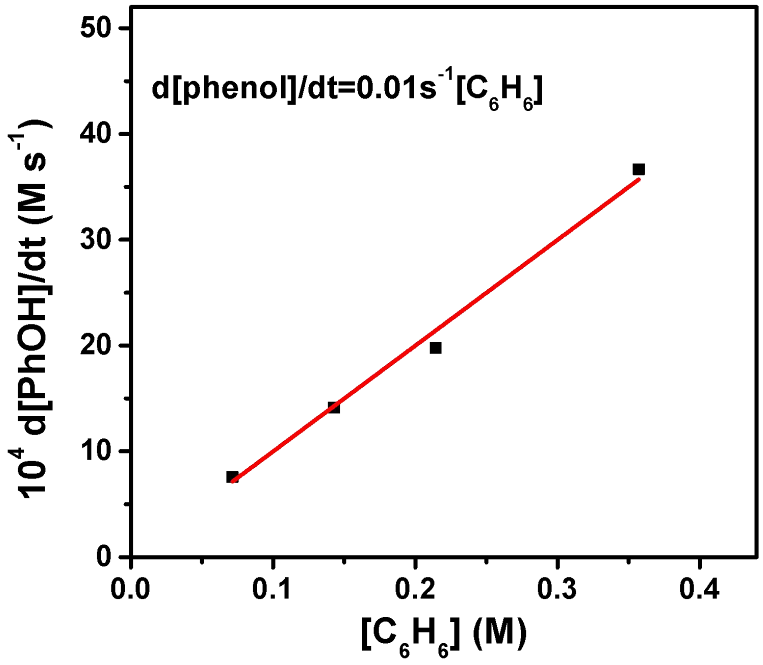

Supplementary Figure 32 | Linear plot of the reaction rate (d[PhOH]/dt) vs. centration of benzene. Reaction conditions: benzene (1 ~ 5 mmol), aqueous H_2O_2 (30%, 5 mmol), VSZ-5 (0.1 g), H_2SO_4 (0.15 g), CH_3CN (14 mL), 80 °C, 30 s. d[PhOH]/dt was calculated from [PhOH]/t at 30 s (Supplementary Fig. 23) by assuming that the kinetic curve belongs to a first-order equation.

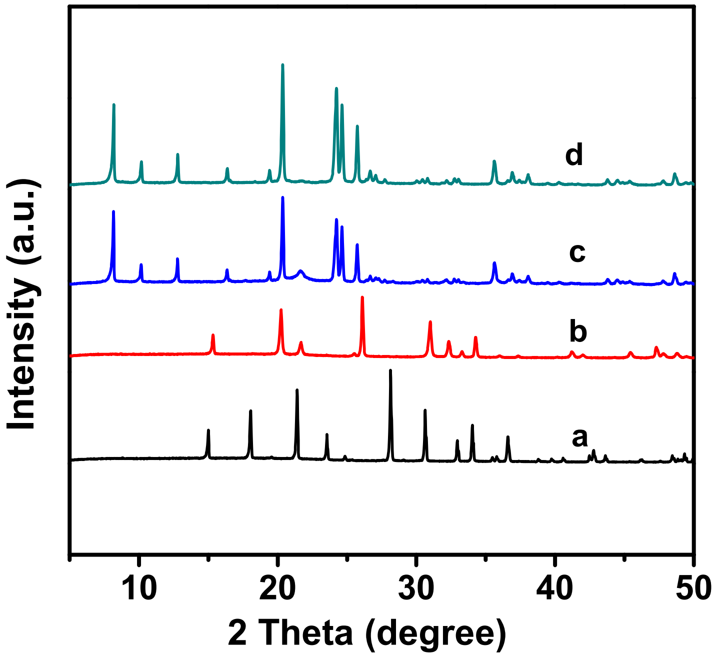

Supplementary Figure 33 | XRD patterns. (a) NH_4VO_3, (b) V_2O_5, (c) V_2O_5@Si-ZSM-22 and (d) V-AlSi-ZSM-22.

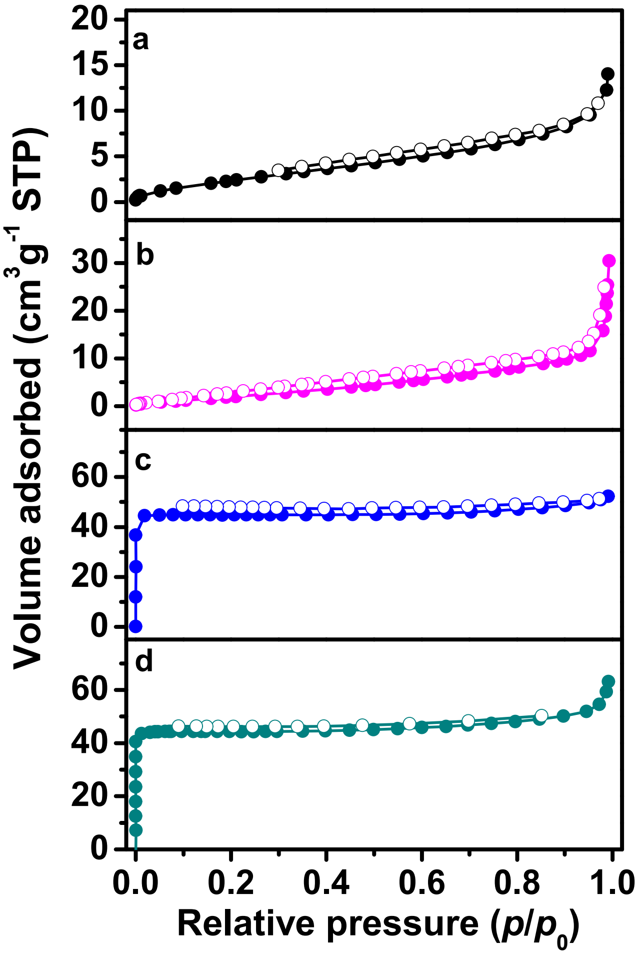

Supplementary Figure 34 | N_2 sorption isotherms of control samples. (a) NH_4VO_3, (b) V_2O_5, (c) V_2O_5@Si-ZSM-22 and (d) V-AlSi-ZSM-22.

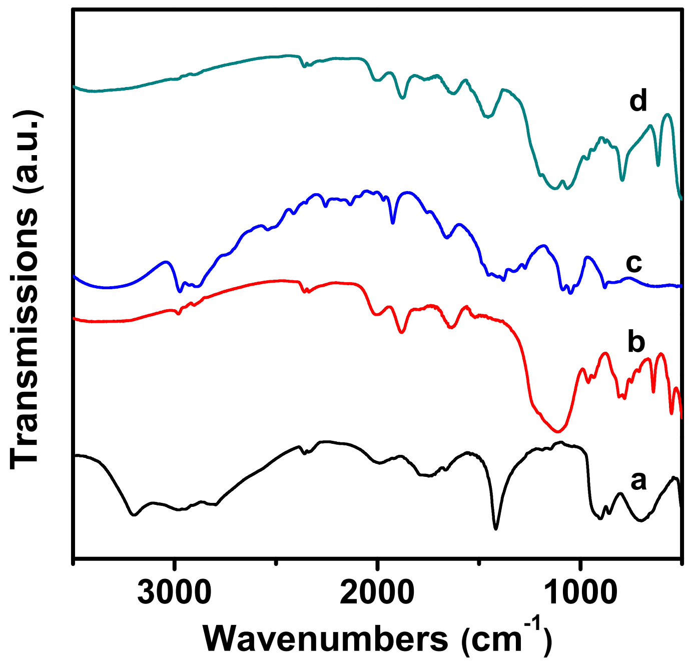

Supplementary Figure 35 | FTIR spectra. (a) NH_4VO_3, (b) V_2O_5, (c) V_2O_5@Si-ZSM-22 and (d) V-AlSi-ZSM-22.

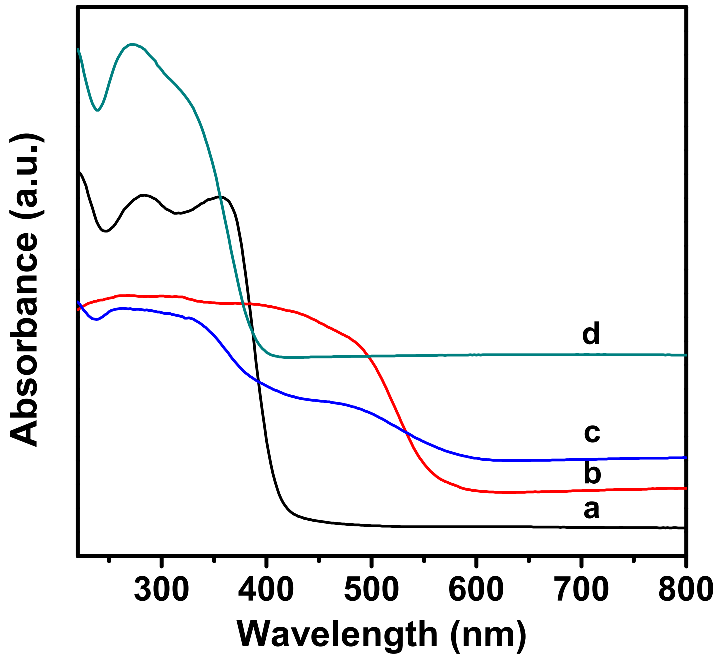

Supplementary Figure 36 | UV spectra. (a) NH_4VO_3, (b) V_2O_5, (c) V_2O_5@Si-ZSM-22 and (d) V-AlSi-ZSM-22.

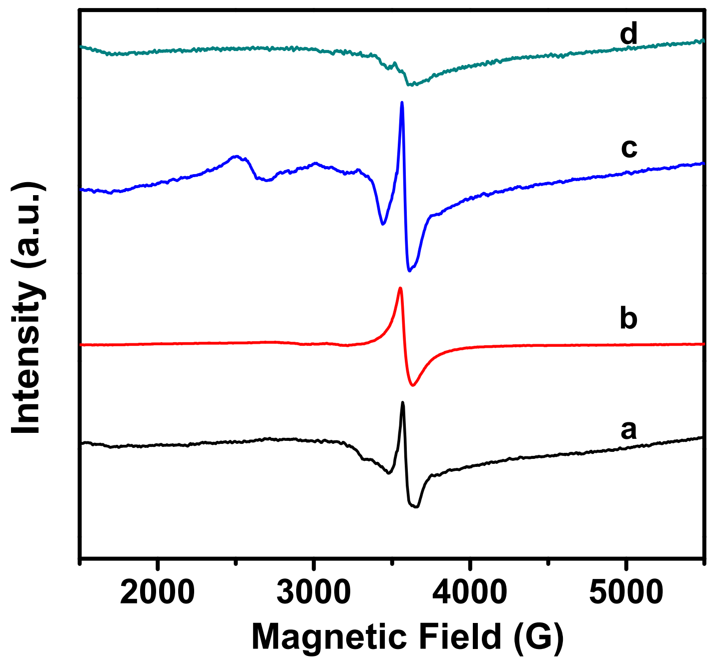

Supplementary Figure 37 | ESR spectra. (a) NH_4VO_3, (b) V_2O_5, (c) V_2O_5@Si-ZSM-22 and (d) V-AlSi-ZSM-22.

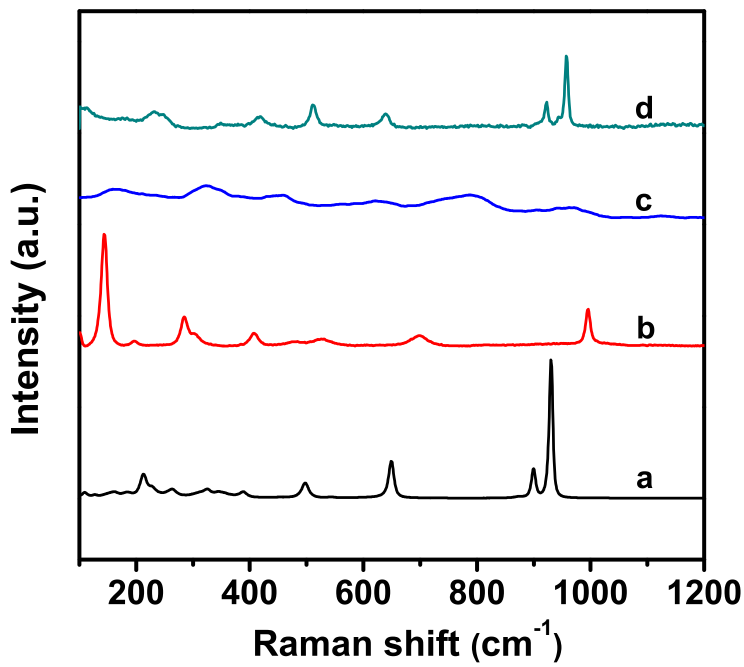

Supplementary Figure 38 | Raman spectra. (a) NH_4VO_3, (b) V_2O_5, (c) V_2O_5@Si-ZSM-22 and (d) V-AlSi-ZSM-22.

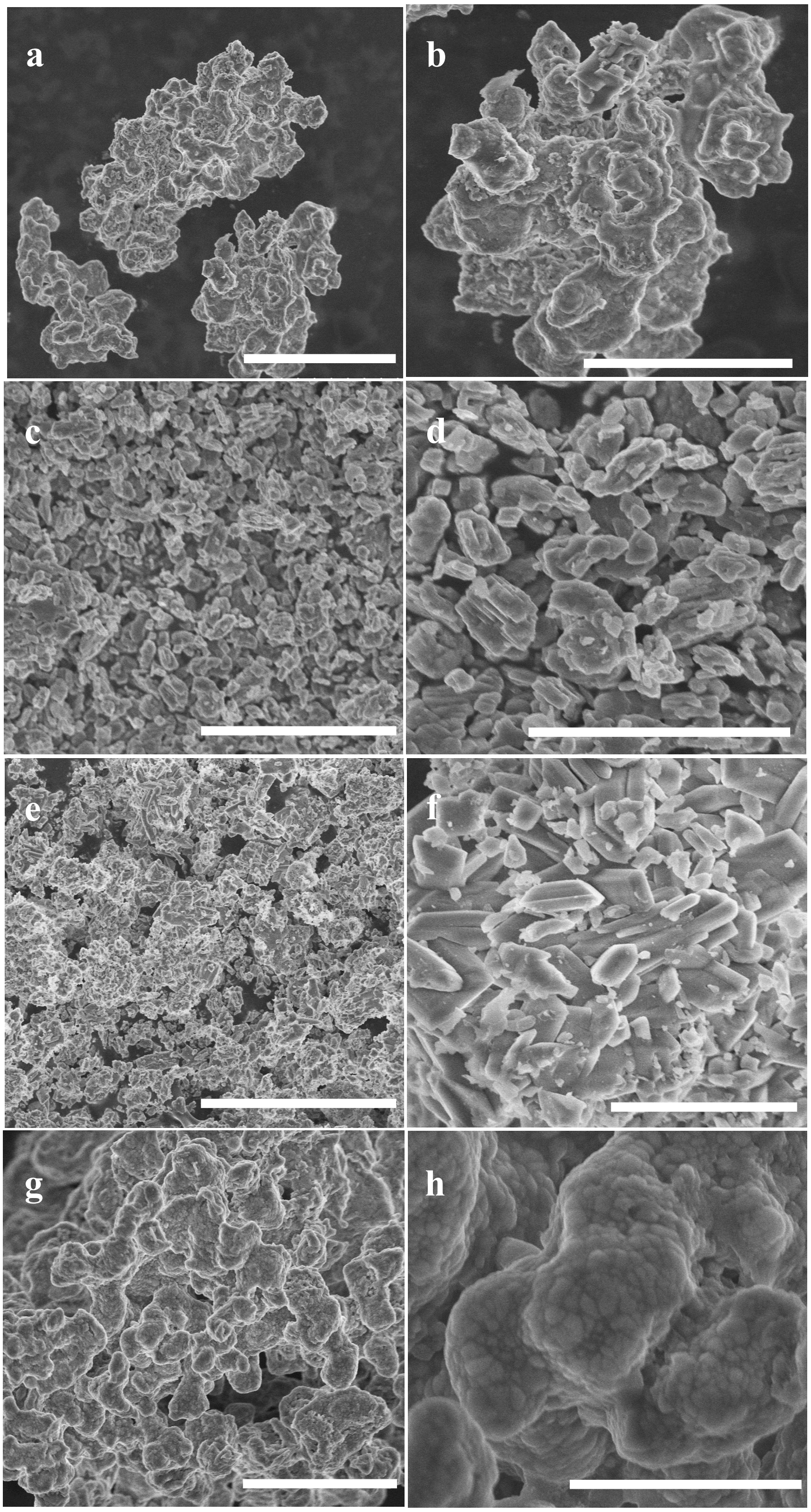

Supplementary Figure 39 | SEM images. (a, b) NH_4VO_3, (c, d) V_2O_5, (e, f) V_2O_5@Si-ZSM-22 and (g, h) V-AlSi-ZSM-22.Scale bars, 20 μm (a), 10 μm (b), 10 μm (c), 5 μm (d), 30 μm (e), 5 μm (f), 10 μm (g), 3 μm (h).

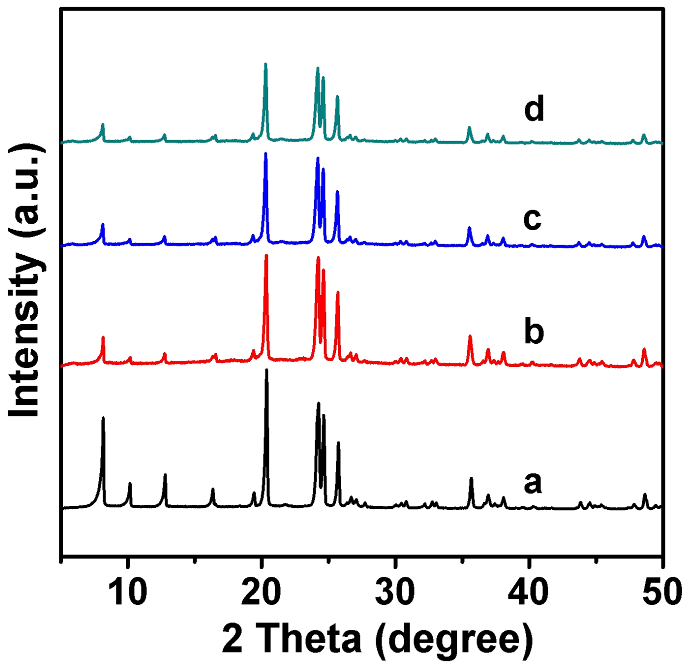

Supplementary Figure 40 | XRD patterns. (a) VSZ-5, (b) VSZ-5(m), (c) VSZ-5(r1) and (d) VSZ-5(r6).

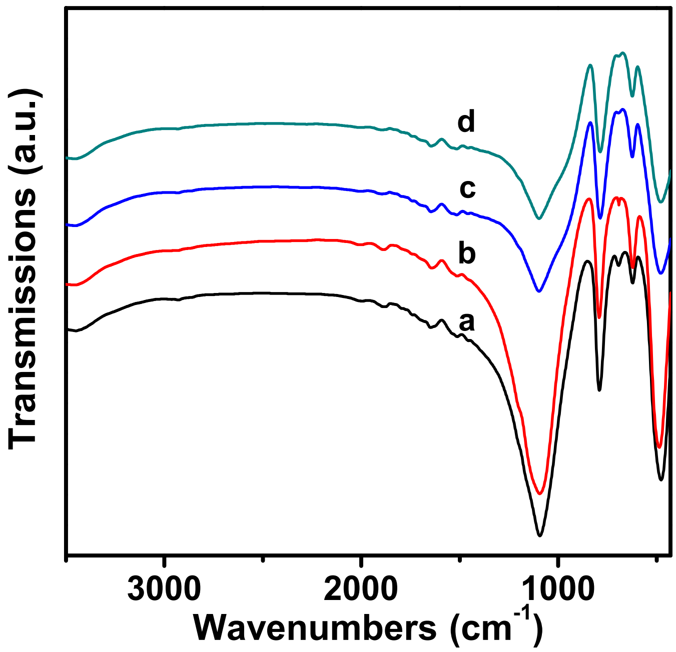

Supplementary Figure 41 | FTIR spectra. (a) VSZ-5, (b) VSZ-5(m), (c) VSZ-5(r1) and (d) VSZ-5(r6).

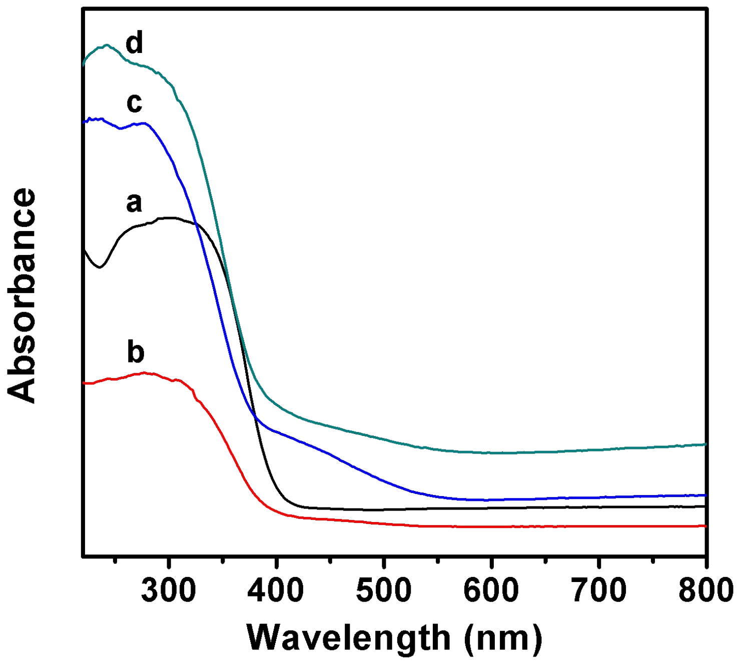

Supplementary Figure 42 | UV-vis spectra. (a) VSZ-5, (b) VSZ-5(m), (c) VSZ-5(r1) and (d) VSZ-5(r6).

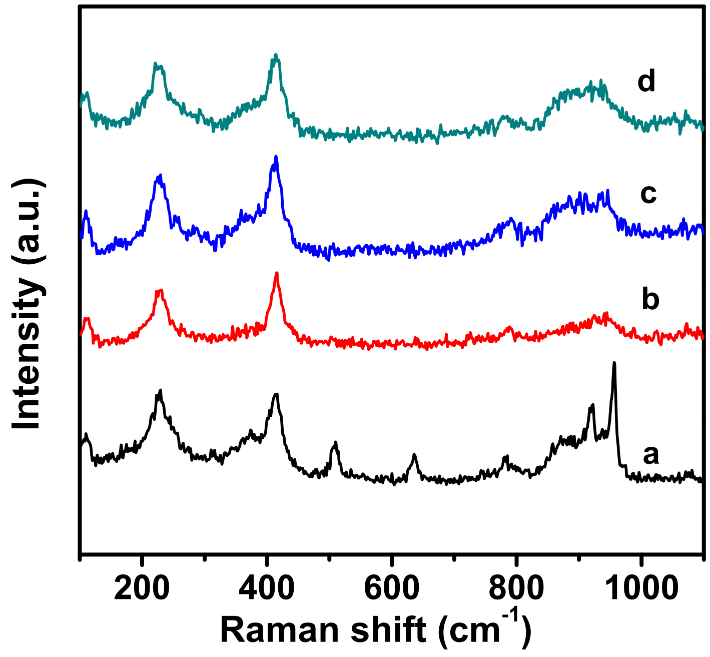

Supplementary Figure 43 | Raman spectra. (a) VSZ-5, (b) VSZ-5(m), (c) VSZ-5(r1) and (d) VSZ-5(r6).

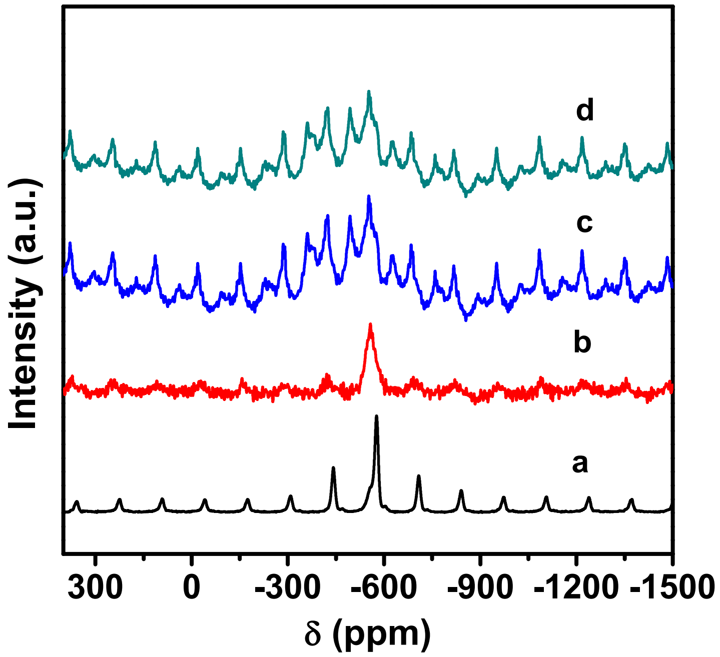

Supplementary Figure 44 | ^{51}V MAS NMR spectra. (a) VSZ-5, (b) VSZ-5(m), (c) VSZ-5(r1) and (d) VSZ-5(r6).

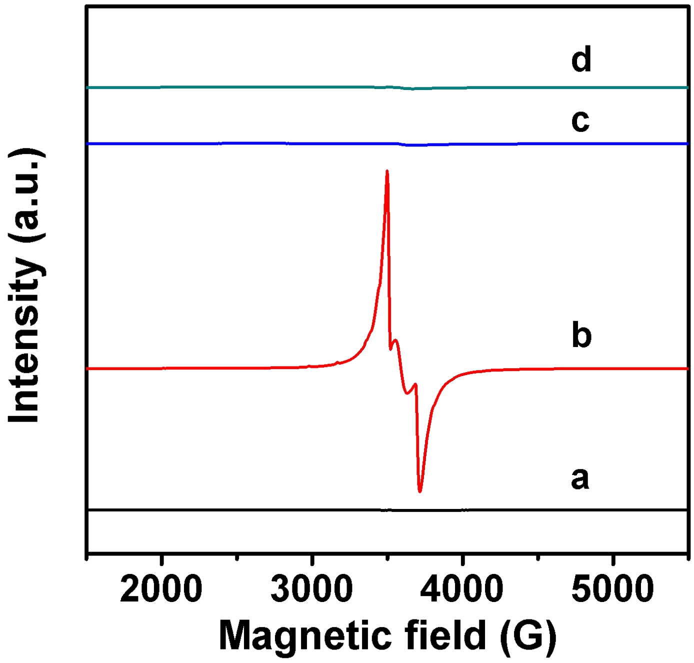

Supplementary Figure 45 | ESR spectra. (a) VSZ-5, (b) VSZ-5(m), (c) VSZ-5(r1) and (d) VSZ-5(r6).

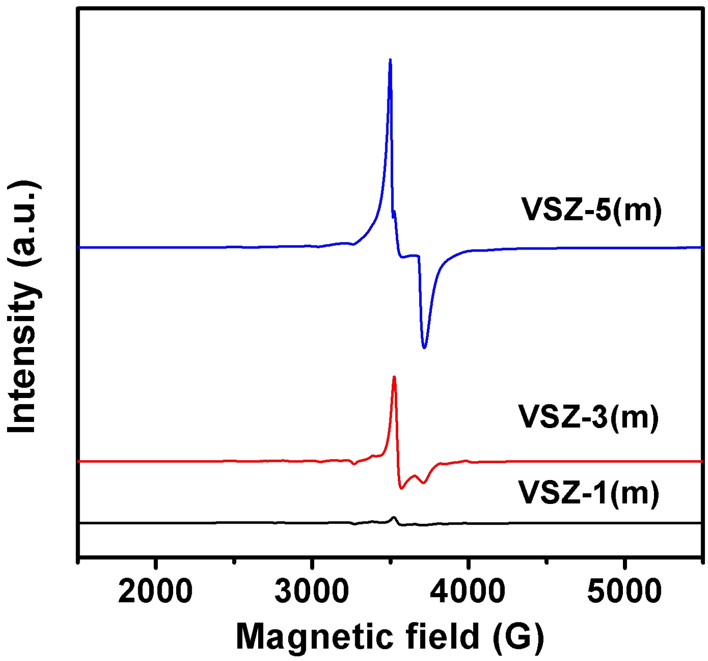

Supplementary Figure 46 | ESR spectra. VSZ-1(m), (b) VSZ-3(m) and (c) VSZ-5(m). Two intermediate samples of VSZ-1(m) and VSZ-3(m) (corresponding fresh catalysts are VSZ-1 and VSZ-3 respectively, in which 1 and 3 stand for 100×[V/Si molar ratio in the gel]) were collected by using the procedure similar to the one used to collect VSZ-5(m). ESR spectra of VSZ-1(m) and VSZ-3(m) were measured and compared with that of VSZ-5(m). All the three samples exhibited apparent ESR signals derived from the formation of V(IV) species. Their peak intensities are in the sequence of VSZ-1(m)<VSZ-3(m)<VSZ-5(m). The ESR observation suggests that more and more V(IV) species were formed as the total V content increased in the catalysts VSZ-1, VSZ-3 and VSZ-5, in line with their activity sequence for the hydroxylation of benzene (Supplementary Table 4, entry 4 (9.6%), 6 (17.6%) and 7(30.8%)) and toluene (Supplementary Table 5, entry 3 (9.8%), 4 (13.4%), and 5 (26.2%)). This comparison reflects a positive correlation between the number of the V(IV) species and the reactivity.

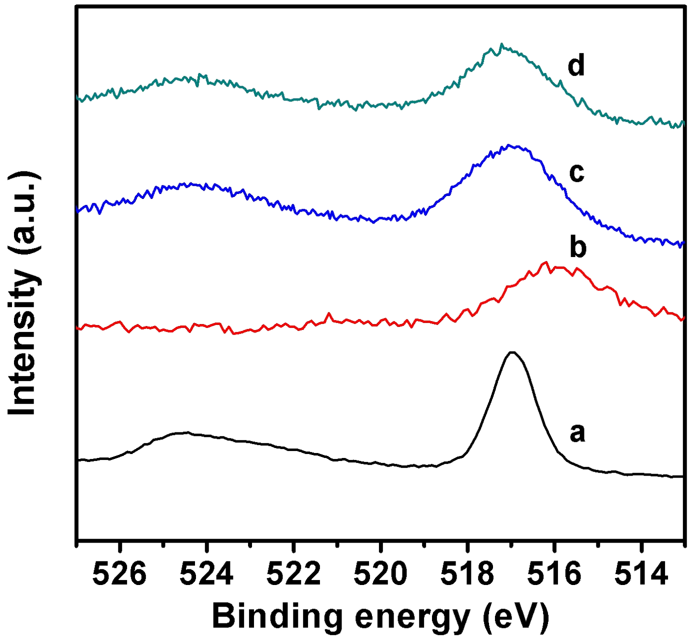

Supplementary Figure 47 | V2p XPS spectra. (a) VSZ-5, (b) VSZ-5(m), (c) VSZ-5(r1) and (d) VSZ-5(r6).

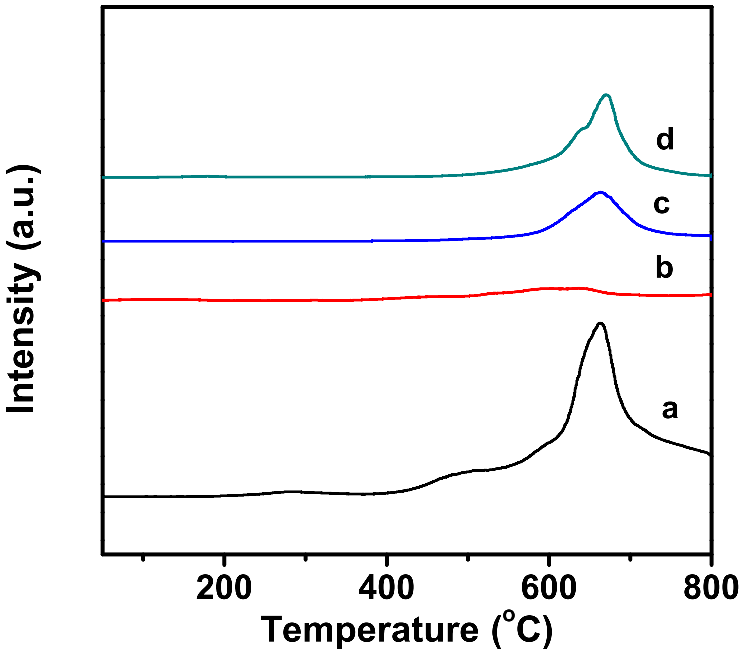

Supplementary Figure 48 | H_2-TPR profiles. (a) VSZ-5, (b) VSZ-5(m), (c) VSZ-5(r1) and (d) VSZ-5(r6).

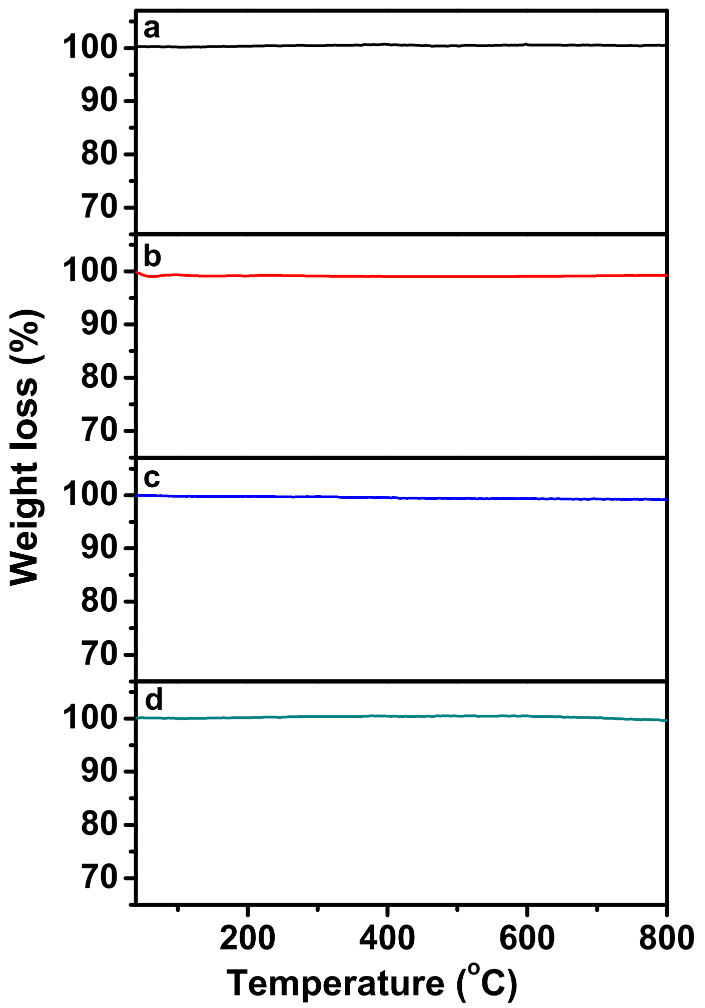

Supplementary Figure 49 | TG curves. (a) VSZ-5, (b) VSZ-5(m), (c) VSZ-5(r1) and (d) VSZ-5(r6).

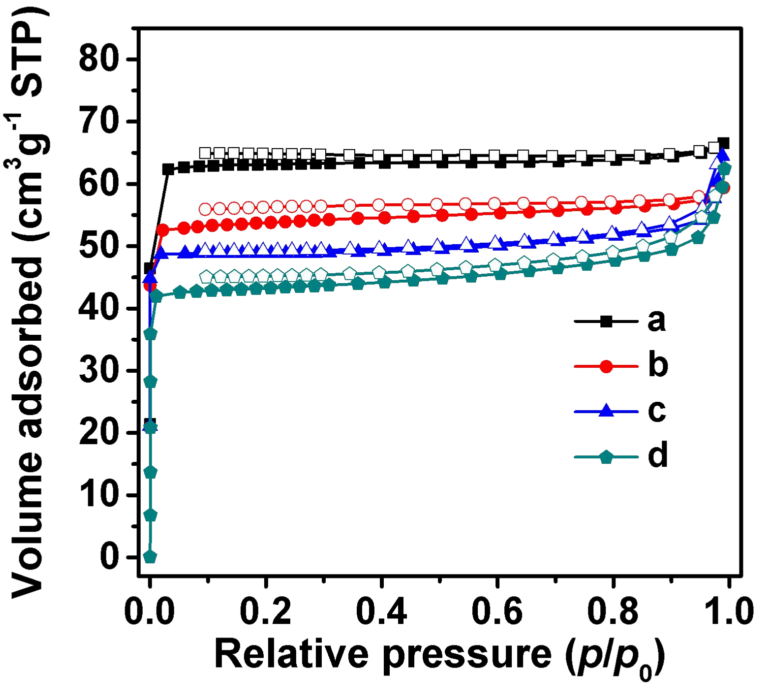

Supplementary Figure 50 | N_2 sorption isotherms. (a) VSZ-5, (b) VSZ-5(m), (c) VSZ-5(r1) and (d) VSZ-5(r6).

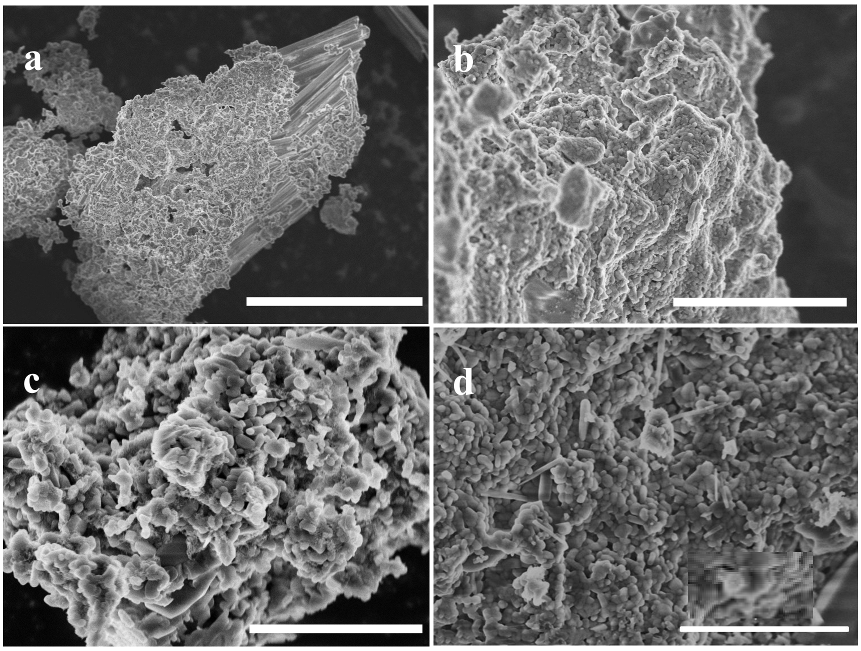

Supplementary Figure 51 | SEM images. (a) VSZ-5, (b) VSZ-5(m), (c) VSZ-5(r1) and (d) VSZ-5(r6). Scale bar, 50 µm (a), 10 µm (b), 5 µm (c), 5 µm (d).

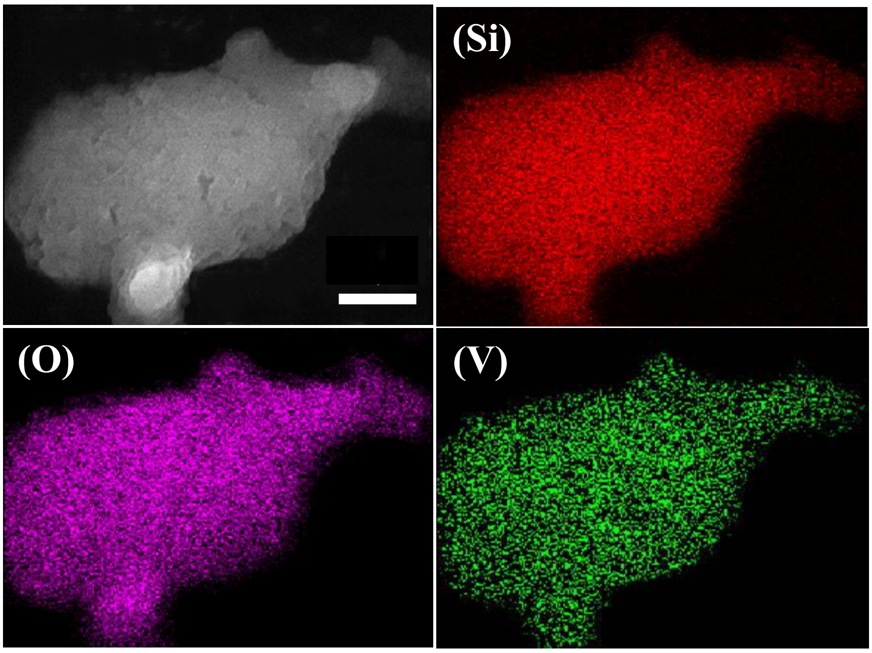

Supplementary Figure 52 | elemental mapping analysis. SEM image and energy-dispersive X-ray spectrometry (EDS) elemental mapping images (Si, O and V element) of VSZ-5(r1). Scale bar, 25 μm.

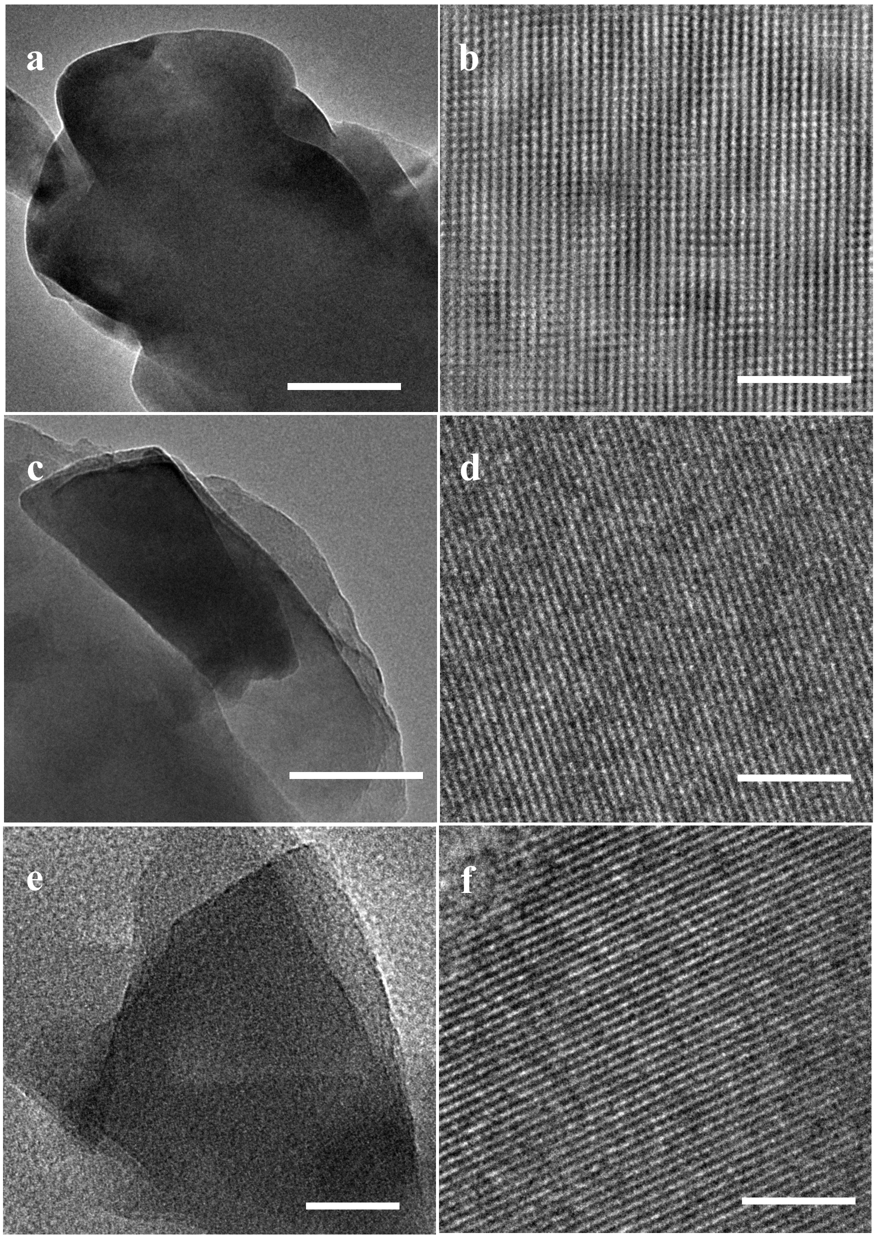

Supplementary Figure 53 | TEM images. (a, b) VSZ-5(m), (c, d) VSZ-5(r1) and (e, f) VSZ-5(r6). Scale bars, 200 nm (a), 5 nm (b), 100 nm (c), 5 nm (d), 20 nm (e), 5 nm (f).

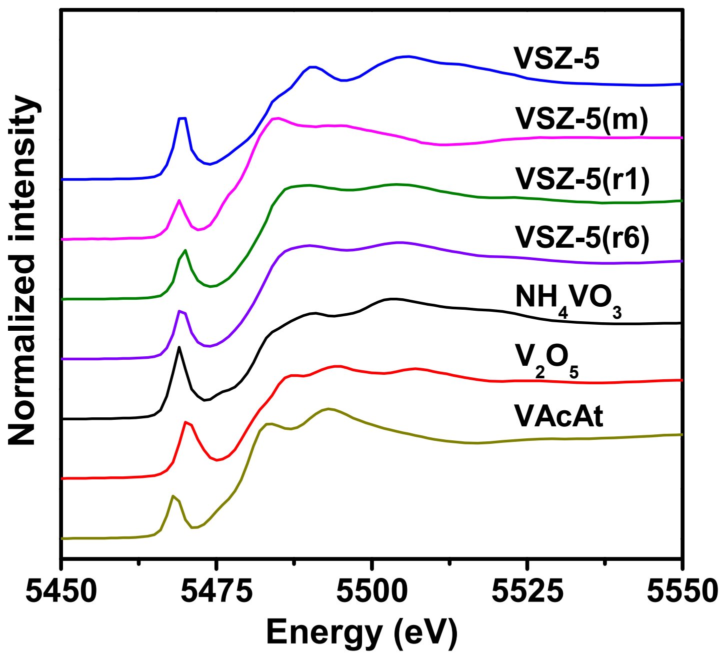

Supplementary Figure 54 | X-ray absorption near edge structure (XANES) of V K-edge for VSZ-5, VSZ-5(m), VSZ-5(r1) and VSZ-5(r6) samples with the reference materials NH_4VO_3, V_2O_5 and VAcAt (vanadium acetylacetonate).

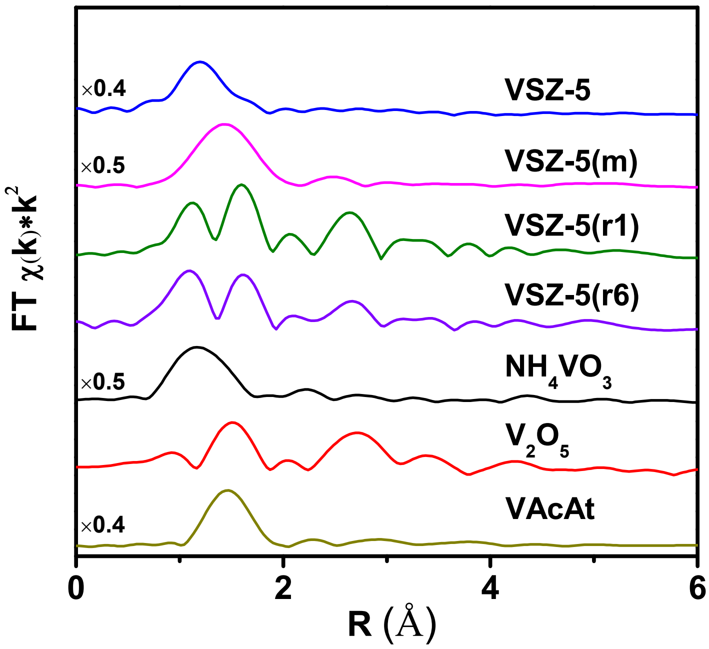

Supplementary Figure 55 | The k^2-weighted Fourier transform spectra derived from EXAFS for VSZ-5, VSZ-5(m), VSZ-5(r1) and VSZ-5(r6) samples with the reference materials NH_4VO_3, V_2O_5 and VAcAt (vanadium acetylacetonate).

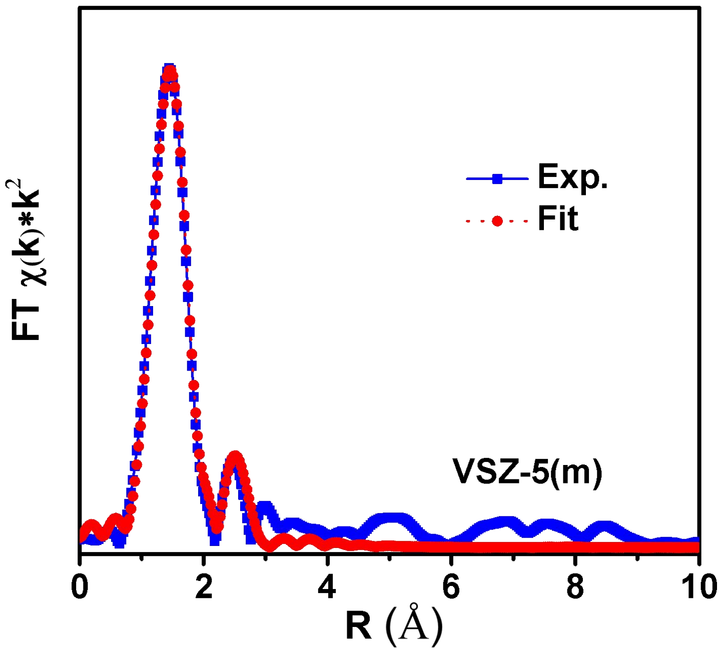

Supplementary Figure 56 | FT-EXAFS curves. The experimental data and the fit for VSZ-5(m).

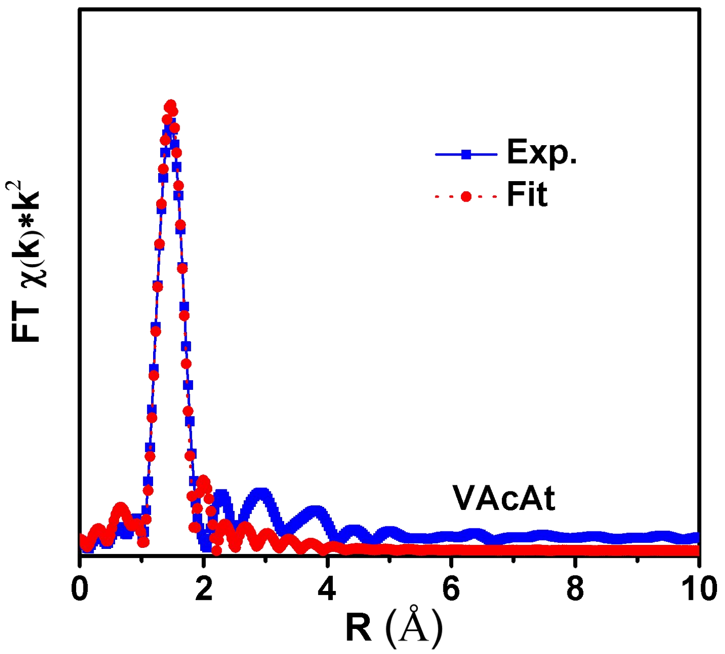

Supplementary Figure 57 | FT-EXAFS curves. The experimental data and the fit for VAcAt.

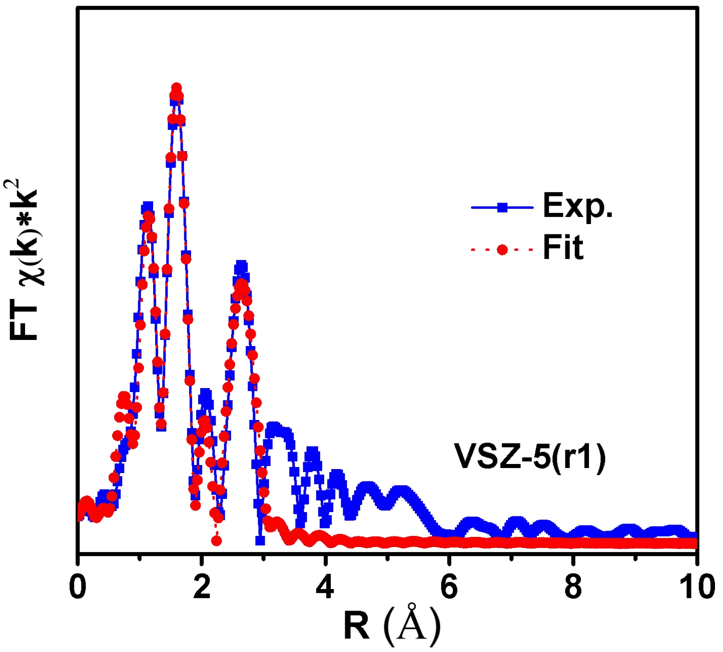

Supplementary Figure 58 | FT-EXAFS curves. The experimental data and the fit for VSZ-5(r1).

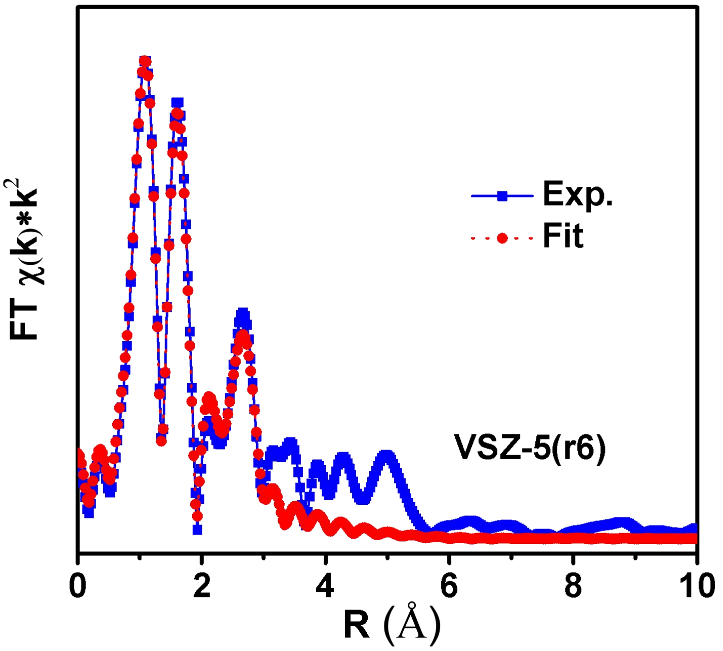

Supplementary Figure 59 | FT-EXAFS curves. The experimental data and the fit for VSZ-5(r6).

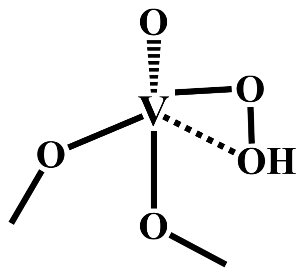

Supplementary Figure 60 | Proposed umbrella model of the V species in VSZ-5(r1) and VSZ-5(r6). These V species are in the high covalent state of V(V), as confirmed by the ESR (Supplementary Fig. 45), XPS (Supplementary Fig. 47), H_2-TPR (Supplementary Fig. 48) and XANES analyses (Supplementary Fig. 54). The adsorption band above 400 nm in the UV-vis spectrum indicates the formation of partially octahedral V(V) species due to the water coordination (Supplementary Fig. 42). The peak around 915 cm^{-1} in the Raman spectrum (Supplementary Fig. 43) implies the formation of O-O structure, and the peak located at 950 is attributable to the V=O. The signal at 493 ppm in the ^{51}V NMR spectrum (Supplementary Fig. 44) suggests the formation of new type V species, which are different from the one in the fresh VSZ-5. This phenomenon also excludes the formation pyramidal V species that were normally found in many V-containing catalysts[1,2]. XAFS analysis (Supplementary Figs 54, 55 and 58 and Supplementary Table 3) revealed that VSZ-5(r1) contained V(V) species, in which there are one short bond (V=O, 1.66 Å), 2.2 long bonds (V–O, 1.92 Å) and one much long bond (V–O, 2.86 Å). Based on above analyses, the most probable V species of VSZ-5(r1) are the umbrella model. The recovered catalyst after 6th run, VSZ-5(r6), demonstrated almost the same structure as VSZ-5(r1) (Supplementary Figs 40-55 and 59 and Supplementary Table 3), indicating that they exhibit the similar structure.

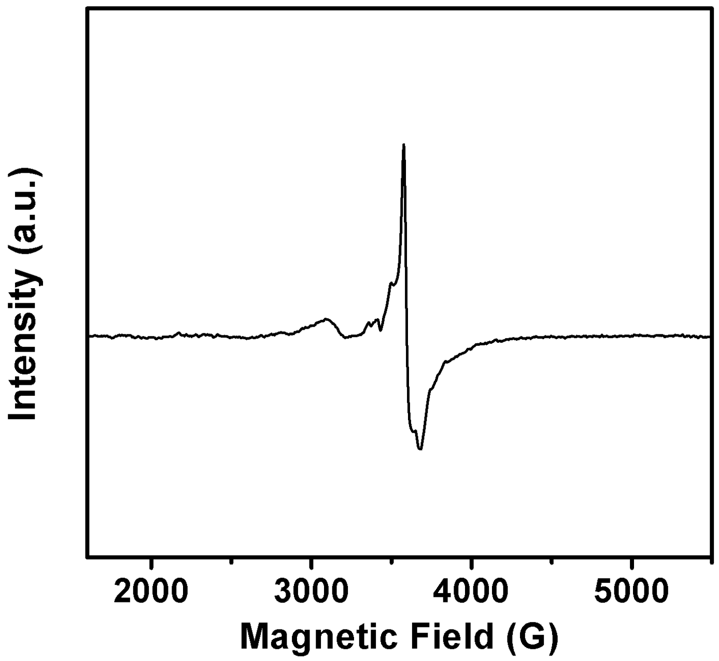

Supplementary Figure 61 | ESR spectrum of VSZ-5(r1)-as.

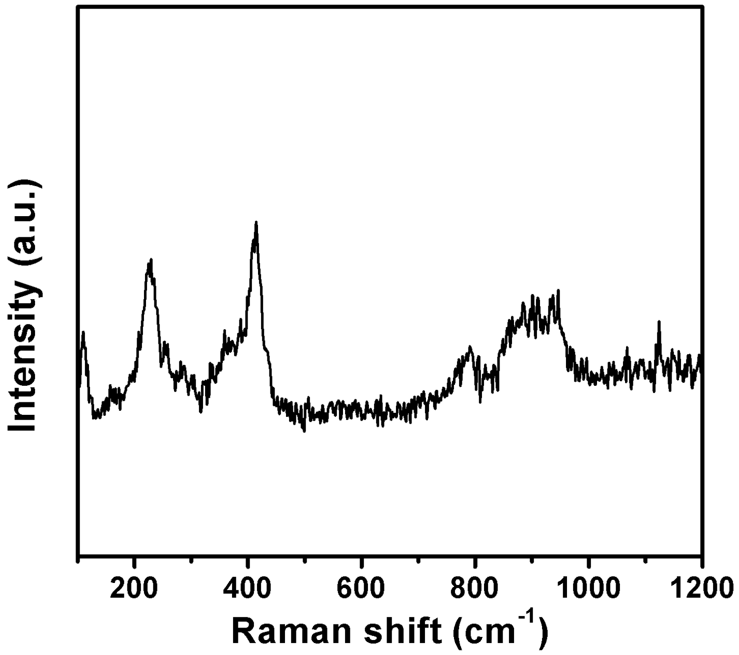

Supplementary Figure 62 | Raman spectrum of VSZ-5(r1)-as.

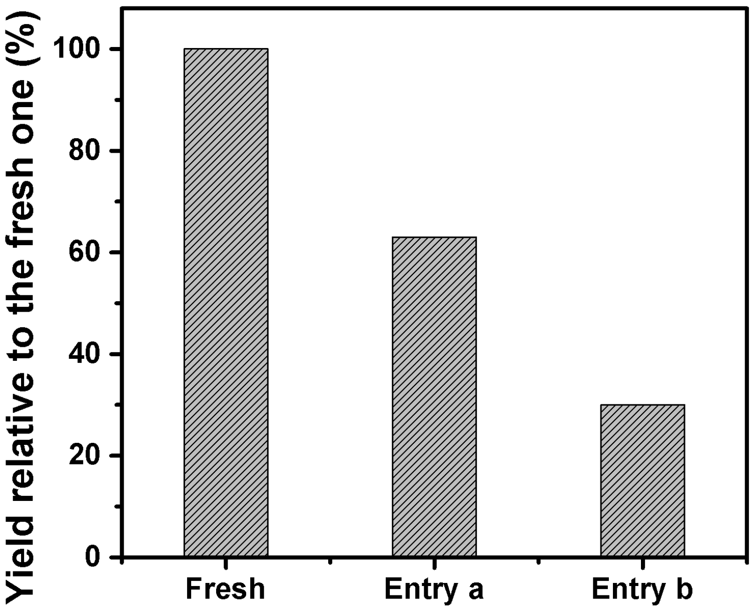

Supplementary Figure 63 | Direct hydroxylation of toluene catalyzed by fresh VSZ-5, (entry a) VSZ-5(r1)-as in the presence of H_2SO_4 and (entry b) VSZ-5(r1)-as in the absence of H_2SO_4. Reaction conditions: toluene (5 mmol), aqueous H_2O_2 (30%, 5 mmol), catalyst (0.1 g), H_2SO_4 (0.15 g), CH_3CN (12 mL), 80 °C, 30 s.

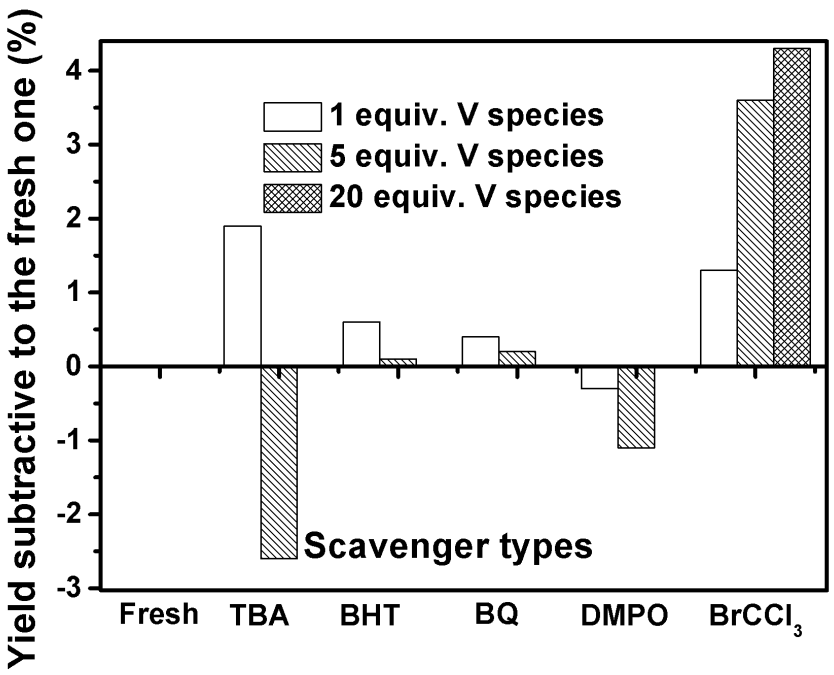

Supplementary Figure 64 | Radical scavenger tests. VSZ-5 catalyzed direct hydroxylation of toluene in the presence of tert-butyl alcohol (TBA, scavenger for hydroxyl radicals), butylated hydroxytoluene (BHT, scavenger for superoxide radicals), benzoquinone (BQ, scavenger for superoxide radicals), 5,5-Dimethyl-1-pyrroline N-oxide (DMPO, radical trapping reagent for various radicals), or bromotrichloromethane ($BrCCl_3$, scavenger for carbon-centered radicals). Reaction conditions: toluene (5 mmol), aqueous H_2O_2 (30%, 5 mmol), catalyst (0.1 g), H_2SO_4 (0.15 g), CH_3CN (12 mL), 80 °C, 30 s.

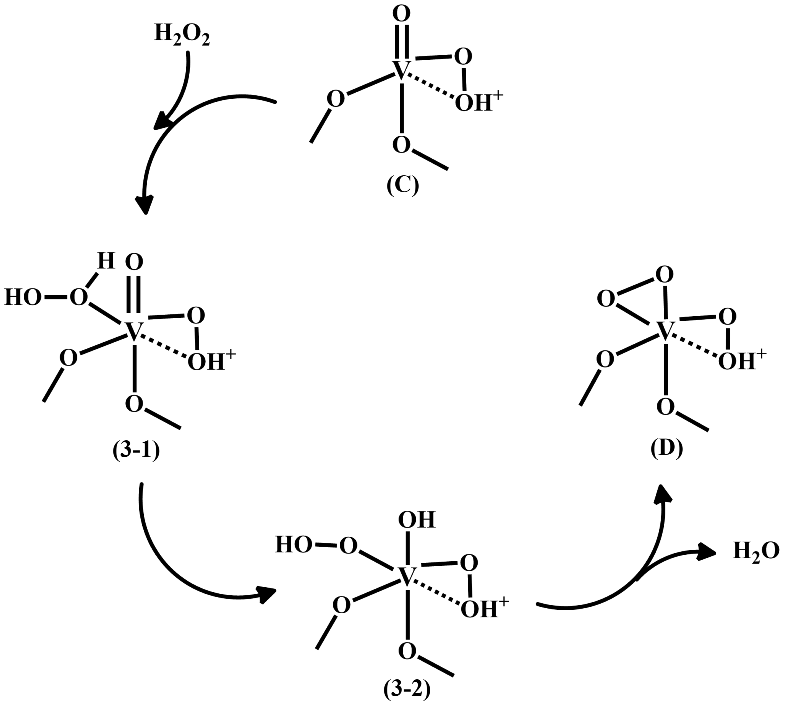

Supplementary Figure 65 | Formation of species (D) through the interaction of species (C) with H_2O_2. It was reported that two possible paths may happen after the steps of the interaction of V^V-peroxo with H_2O_2 and the H-transfer[3-5]: (1) Elimination of H_2O causing the formation of diperoxo-V species; (2) Elimination of HOO· yielding V^{IV} species. Previous work indicated that these two paths had similar activation energy barriers, but formed diperoxo-V species were more stable than V^{IV} species[3,4]. In this work, mechanisms involving free radicals were excluded based on the catalytic performance in the hydroxylation of different arenes (benzene and its derivatives with electron-donating and withdrawing groups), KIE analysis, and radical scavenger tests. Therefore, we propose that the elimination of H_2O is the most probable way in the variation from species (**C**) to (**D**).

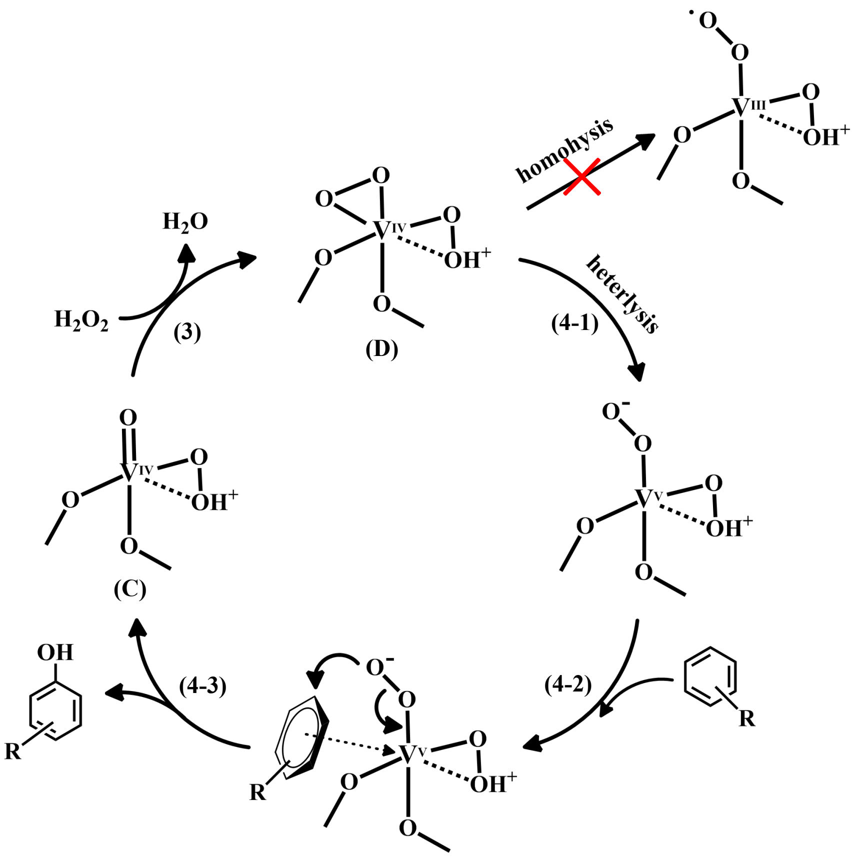

Supplementary Figure 66 | Heterolysis of species (**D**) and successive interaction with arene to produce phenol.

Previously, electrophilic, non-radical aromatic substitution mechanism was proposed for Os, Cu and V based homogeneous systems[6-10]. In these hydroxylation processes, the formed metal-active oxygen species, usually in the form of metal=O, metal-O-O· and metal bis(μ-oxo), were involved in an electrophilic attack on the benzene ring, resulting in high activity by using the mono-substituted benzenes with electron-donating groups such as anisole and phenol. By contrast, apparent high activity in the hydroxylation of benzene and suppression of the activity in the hydroxylation of anisole and phenol was observed using the VSZ-5 catalyst. Besides, for aryl halides substrates with electron-withdrawing groups, the high activity and chemo-selectivity were similarly obtained in the current study, which was rarely achieved in those previous electrophilic reactions. These phenomena suggest that our V-based hydroxylation process is different from those previous systems involving electrophilic attack[6-10].

V-catalyzed oxidations usually involve the formation of peroxo vanadyl complexes[3,4,11-14]. In most cases, hydroperoxyl (HOO·) and hydroxyl (HO·) radicals were active sites[3,4,12,13]. Alternatively, oxygen transformation from V-peroxo complex to arenes was also achieved through the formation of metal-oxygen active sites, including both V-based electrophilic radicals and nucleophilic agents[4,11-15]. It was reported that the reaction of V(V)-peroxo species with arene to produce phenol may undergo either homolysis or heterolysis of V-O bond of the V-peroxo group[4,12-14]. The heterolysis of V^{V}-peroxo species will cause the formation of V^{VI}-O-O^{-} and then V^{V}-O^{+}-O^{-} species. It is more likely that V^{V}-peroxo species undergoes a homolysis of V-O of the V-peroxo to V^{IV}-O-O· radicals[12-14].

In this work, structure characterization of the fresh VSZ-5 and the intermediate VSZ-5(m) indicated that the tetrahedral metavanadate V(V) species $(VO_3)_n^{n-}$ in the fresh catalyst changed to V(IV) species (**B**) after the addition of H_2SO_4. Previous studies of the behavior of V species in the presence of H_2O_2 suggested that the species (**B**) is readily converted into (**C**) [3-5,11-14]. The observed umbrella model V species in the recovered catalysts VSZ-5(r1) and VSZ-5(r6) as well as the characterization of VSZ-5(r1)-as support the formation of this V(IV)-based peroxo compound during the reaction. The structure characterization and activity assess of VSZ-5(r1) and VSZ-5(r1)-as implies that the arene was oxidized by the active metal-oxygen sites derived from species (**C**) in the presence of H_2O_2. The interaction of species (C) with H_2O_2 to create species (D) is preferred in a non-radical hydroxylation reaction (Supplementary Fig. 65)[3,4]. The formed diperoxo group in species (D) is a highly active oxygen transfer agent to initiate the ring hydroxylation of arenes[3-5,11-14]. The V(IV)-based peroxo in this work is structural similar to the above mentioned previous V(V)-based peroxo, but with clear difference in V valence, and thus leads to a different catalytic behavior. The homolytic cleavage of the V-O bond of V^{IV}-peroxo species will cause the formation of V^{III}-O-O· radicals (with further lower valence), whereas heterolysis will produce the V^{V}-O-O^{-} species (Supplementary Fig. 66). Based on 1) the radical hydroxylation route is experimentally excluded; 2) the switch between V^{IV} and V^{V} is more energy favorable for the H_2O_2-mediated

hydroxylation[3,4,12-14]; 3) metal-oxygen species have also been reported to be involved in some nucleophilic attack processes (despite that they are more usually considered as electrophilic agents)[4,11-15], we tentatively propose that the heterolysis of V^{IV}-peroxo species into V^{V}-O-O^{-} species is mostly the possible pathways in the oxidation of benzene ring (Supplementary Fig. 66). Such special behavior is attributable to the formation of V(IV) peroxo compounds that lead to an unusual oxidation process. The V^{V} cation of V^{V}-O-O^{-} is a Lewis acidic site and able to interact with the negative π-system of the benzene ring *via* polarization. Such polarization interaction promotes the approaching and adsorption of arene, and the successive nucleophilic attack of O^{-} species on the C and H to form a transition complex, the cleavage of which produces phenol and regenerates species (**C**). In a traditional electrophilic substitution reaction of arenes, the following two steps are crucial: 1) an electrophilic agent attacks the C atom to form carbocation or a transition complex; 2) a nucleophilic agent attacks the formed carbocation or transition complex to afford the product. Similarly, the interaction of V^{V}-O-O^{-} with benzene ring involves the electrophilic attack of V^{V} cation on the benzene ring and the nucleophilic attack of O^{-} species on the C and H (this oxidation step is an oxygen transfer process, in which the O simultaneously interacts with C and H, enabling the H transfer from C to O). Owing to that, the reaction still involves the electrophilic attack of V^{V} cation on the C in benzene ring, and *o*- and *p*-products were obtained. In the presence of an electro-denoting group, the nucleophilic attack of O^{-} species on the electron-enriched C atom may be hindered, which decreases the activity in this step. Methoxy and hydroxyl group are electron-donating groups as they have the strong resonance effect (though with the coexistence of weak inductive effect). Thus, the hydroxylation of anisole and phenol is not favored through the proposed mechanism above, in line with the experimental results.

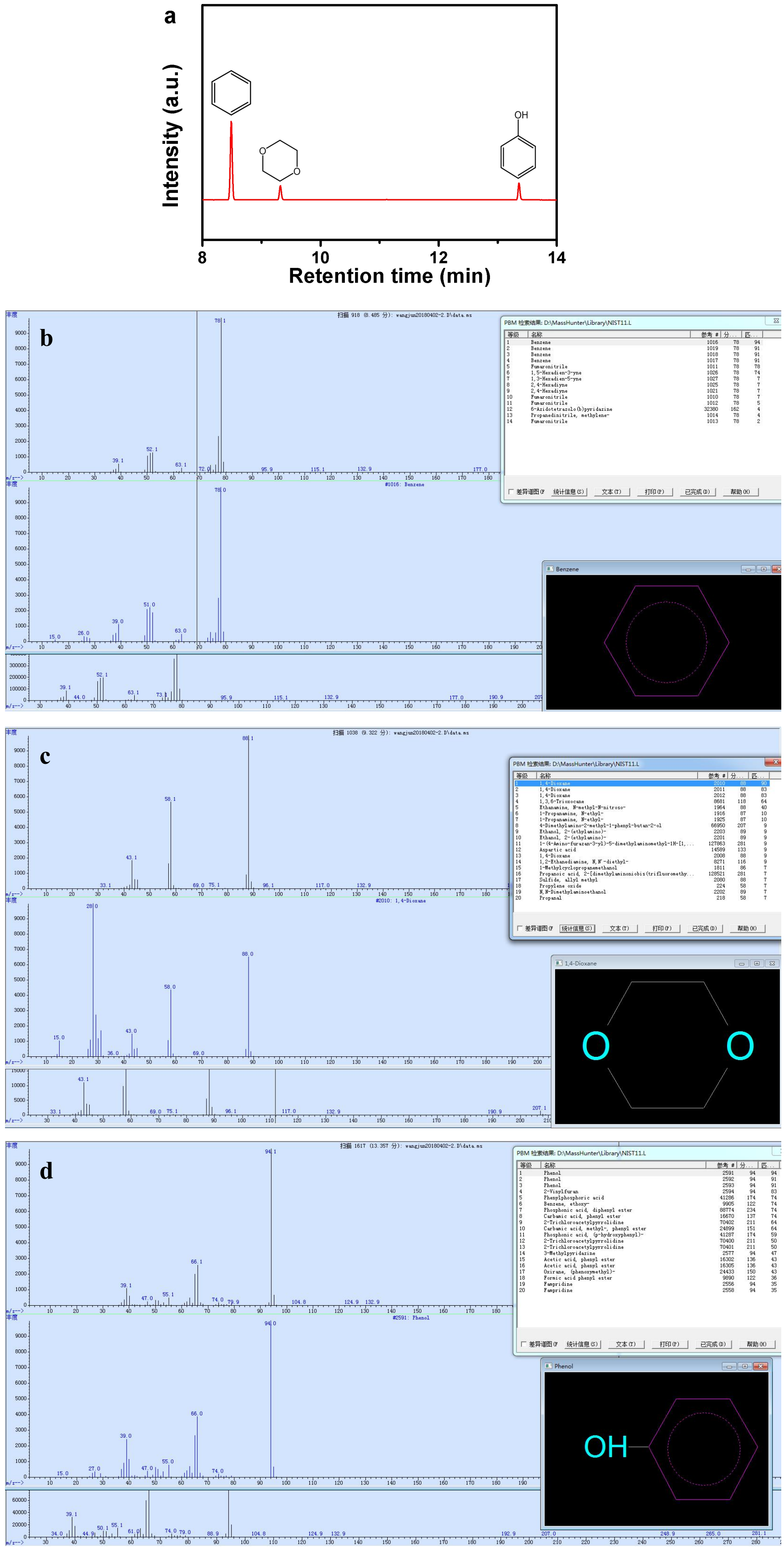

Supplementary Figure 67 | (a) GC and (b-d) GS/MSD spectra of a typical hydroxylation of benzene catalyzed by VSZ-5.

Supplementary Table 1. Textural properties.†

Sample	n_{Si}/n_V		n_{Na}/n_V	S_{BET}	V_p
	gel‡	solid§	solid‖	$(m^2 \cdot g^{-1})$*	$(cm^3\ g^{-1})$¶
Si-ZSM-22	-	-	-	265	0.12
VSZ-1	100	105.2	3.4	242	0.10
VSZ-3	33	35.0	3.2	218	0.096
VSZ-5	20	20.4	2.5	211	0.092
VSZ-10	10	10.4	2.4	95	0.038
VSZ-15	6.7	6.9	1.9	48	0.017
VSZ-20	5	5.2	1.2	16	0.012
VSZ-25	4	4.1	1.1	5	0.007
NH_4VO_3	-	-	-	10	0.020
V_2O_5	-	-	-	4	0.040
V_2O_5@Si-ZSM-22	-	22.9	-	158	0.081
V-AlSi-ZSM-22$^{\Delta}$	20	21.5	2.5	183	0.087
VSZ-5(m)	-	20.7	2.4	198	0.091
VSZ-5(r1)	-	21.2	2.5	156	0.086
VSZ-5(r6)	-	21.7	2.5	145	0.088

†Reaction conditions: toluene (5 mmol), H_2O_2 (30%, 5 mmol), catalyst (0.1 g), CH_3CN (12 mL), H_2SO_4 (0.15 g), 80 °C, 30 s. ‡The molar ratio of Si to V in the gel determined by XRF. §Molar ratio of Si to V in the final solid. ‖Molar ratio of Na to V in the final solid. *Surface area. ¶Total pore volume. $^{\Delta}$Molar ratio of SiO_2 to Al_2O_3=102 in the final solid.

Supplementary Table 2. Unit cell parameters.

Sample	Unite cell parameters†			
	a (Å)	b (Å)	c (Å)	V (Å^3)
Si-ZSM-22	13.832	17.399	5.031	1210.702
VSZ-1	13.844	17.411	5.039	1214.571
VSZ-3	13.851	17.420	5.041	1216.412
VSZ-5	13.855	17.429	5.045	1218.177
VSZ-10	13.853	17.416	5.045	1217.211
VSZ-15	13.851	17.411	5.046	1216.649
VSZ-20	13.840	17.403	5.051	1216.451
VSZ-25	13.828	17.396	5.056	1216.361

†Unit cell parameters are determined by using the MDI Jade software (Jade 7 XRD Pattern processing Software).

Supplementary Table 3. EXAFS parameters.

Sample	Shell	CN	R(Å)	σ^2 (Å^2)	R-factor
VSZ-5	V-O	2.4±0.4	1.66±0.02	0.0026±0.0008	0.013
	V-O	2.0±0.4	1.82±0.02	0.0026±0.0008	
VSZ-5(m)	V-O	1.1±0.2	1.60±0.01	0.0026±0.0012	0.006
	V-O	3.0±0.7	2.02±0.01	0.0146±0.0044	
	V-V	2.0±0.3	3.08±0.01	0.0146±0.0044	
VSZ-5(r1)	V-O	0.9±0.1	1.66±0.01	0.0008±0.0005	0.0015
	V-O	2.2±0.2	1.92±0.01	0.0057±0.0012	
	V-O	1.0±0.2	2.86±0.01	0.0076±0.0021	
	V-V	2.0±0.4	3.12±0.02	0.0076±0.0021	
VSZ-5(r6)	V-O	1.1±0.2	1.65±0.01	0.0024±0.0009	0.020
	V-O	2.8±0.5	1.89±0.02	0.0103±0.0023	
	V-O	1.0±0.1	2.81±0.03	0.0103±0.0023	
	V-V	2.0±0.4	3.08±0.02	0.0103±0.0023	
NH_4VO_3	V-O	2*	1.66±0.02	0.0023±0.0003	0.009
	V-O	2*	1.82±0.02	0.0023±0.0003	
VAcAt	V-O	1*	1.59±0.02	0.0015±0.0006	0.020
	V-O	4*	1.96±0.02	0.0015±0.0006	
V_2O_5	V-O	1*	1.59±0.01	0.0047±0.0012	0.021
	V-O	2*	1.83±0.02	0.0103±0.0019	
	V-O	2*	1.92±0.02	0.0103±0.0019	
	V-O	1*	2.73±0.03	0.0103±0.0019	
	V-V	2*	3.11±0.03	0.0079±0.0012	

CN, coordination number; R, bonding distance; σ^2, Debye-Waller factor; ΔE_0 shift in absorption edge energy

*This value was fixed during EXAFS fitting, based on the known structure of V.

Supplementary Table 4. Direct hydroxylation of benzene with H_2O_2.[†]

Entry	Catalyst	Catalyst weight (g)[‡]	Additive	S (%)[§]	Y (%)[ǀ]	TOF (h^{-1})[*]
1	None	-	-	~0	~0	0
2	None	-	0.15 g H_2SO_4	~0	~0	0
3	Si-ZSM-22	0.1	0.15 g H_2SO_4	51.3	<0.5	-
4	VSZ-1	0.1	0.15 g H_2SO_4	76.9	9.6	3653
5	VSZ-1	0.05	0.15 g H_2SO_4	77.2	7.5	5707
6	VSZ-3	0.1	0.15 g H_2SO_4	94.7	17.6	2105
7	VSZ-5	0.1	0.15 g H_2SO_4	99	30.8	2315
8	VSZ-10	0.1	0.15 g H_2SO_4	95.8	23.2	1386
9	VSZ-15	0.1	0.15 g H_2SO_4	93.5	22.1	943
10	VSZ-20	0.1	0.15 g H_2SO_4	92.8	21.3	566
11	VSZ-25	0.1	0.15 g H_2SO_4	90.2	20.8	462
12	NH_4VO_3	0.005	0.15 g H_2SO_4	81.2	14.8	556
13	V_2O_5	0.008	0.15 g H_2SO_4	76.5	10.6	796
14	V-AlSi-ZSM-22	0.1	0.15 g H_2SO_4	87.1	15.4	1115
15	V_2O_5@Si-ZSM-22	0.1	0.15 g H_2SO_4	81.4	12.1	876

[†]Reaction conditions: benzene (5 mmol), H_2O_2 (30%, 5 mmol), CH_3CN (14 mL), 80 °C, 30 s. [‡]For the entry 11-14, the V mole content in these catalysts was same as the one in 0.1 g VSZ-5. [§]Yield of phenol: [phenol (mol)/initial benzene (mol)]×100. [ǀ]Selectivity for phenol: [phenol (mol)/converted benzene (mol)]×100. [*]Turnover frequency (TOF)=[phenol (mol)/(V (mol)×reaction time (h)].

Supplementary Table 5. Direct hydroxylation of toluene with H_2O_2.[†]

Entry	Catalyst	Catalyst weight (g)[‡]	Solvent	Additive	S (%)[§]	Y (%)[ǀ]	TOF (h^{-1})[*]
1	None	-	CH_3CN	-	~0	~0	0
2	None	-	CH_3CN	0.15 g H_2SO_4	~0	~0	0
3	VSZ-1	0.1	CH_3CN	0.15 g H_2SO_4	91.2	9.8	3730
4	VSZ-3	0.1	CH_3CN	0.15 g H_2SO_4	90.7	13.4	1714
5	VSZ-5	0.1	CH_3CN	0.15 g H_2SO_4	91.8	26.2	1969
6	VSZ-10	0.1	CH_3CN	0.15 g H_2SO_4	90.5	19.6	771
7	VSZ-15	0.1	CH_3CN	0.15 g H_2SO_4	90.1	19.1	510
8	VSZ-20	0.1	CH_3CN	0.15 g H_2SO_4	90.4	16.2	334
9	VSZ-25	0.1	CH_3CN	0.15 g H_2SO_4	87.9	12.8	213
10	NH_4VO_3	0.005	CH_3CN	0.15 g H_2SO_4	76.2	11.6	407
11	V_2O_5	0.008	CH_3CN	0.15 g H_2SO_4	70.8	8.3	283
12	V-AlSi-ZSM-22	0.1	CH_3CN	0.15 g H_2SO_4	83.9	11.9	894
13	V_2O_5@Si-ZSM-22	0.1	CH_3CN	0.15 g H_2SO_4	79.6	9.7	729
14	VSZ-5	0.1	CH_3CN	-	56.9	1.8	135
15	VSZ-5	0.1	H_2O	0.15 g H_2SO_4	~0	~0	0
16	VSZ-5	0.1	CH_3OH	0.15 g H_2SO_4	~0	~0	0
17	VSZ-5	0.1	CH_3CH_2OH	0.15 g H_2SO_4	~0	~0	0
18	VSZ-5	0.1	DMF	0.15 g H_2SO_4	~0	~0	0
19	VSZ-5	0.1	C_6H_5CN	0.15 g H_2SO_4	~0	~0	0
20	VSZ-5	0.1	CH_3COCH_3	0.15 g H_2SO_4	~0	~0	0
21	VSZ-5	0.1	DMSO	0.15 g H_2SO_4	~0	~0	0
22	VSZ-5	0.1	CH_3CN	V_C	86.3	1.2	90
23	VSZ-5	0.1	CH_3CN	HAc	72.5	1.4	105
24	VSZ-5	0.1	CH_3CN	$HClO_4$	~0	~0	0
25	VSZ-5	0.1	CH_3CN	HCl	~0	~0	0
26	VSZ-5	0.1	CH_3CN	CF_3SO_3H	24.8	1.4	105
27	VSZ-5(m)	0.1	CH_3CN	0.15 g H_2SO_4	91.2	17.1	1285

[†]Reaction conditions: toluene (5 mmol), H_2O_2 (30%, 5 mmol), CH_3CN (12 mL), 80 °C, 30 s. [‡]For the entry 2-6, the V mole content in these catalysts was same as the one in 0.1 g VSZ-5. [§]Yield of cresols: [cresols (mol)/initial toluene (mol)]×100; equal molar ratio of *o*- and *p*-cresol forms while no *m*-cresol is detected. [ǀ]Selectivity for cresols: [cresols (mol)/converted toluene (mol)]×100. [*]Turnover frequency (TOF)=[cresols (mol)/(V (mol)×reaction time (h)].

Supplementary Table 6. Direct hydroxylation of benzene with H_2O_2 catalyzed by different catalysts.

Catalyst	Catalytic type	C/O/S†	Reaction conditions	R/C‡	Ys/Yo§ (%)	TON	TOF (h^{-1})	Mechanism	Ref.
$[Ni^{II}(tepa)]^{2+}$	homogeneous	1/50000/10000	CH_3CN, 60 °C, 216 h	99/1	7.5/1.5	749	3.5	Radical	8
$[Cu_2(\mu\text{-OH})(6\text{-hpa})]^{3+}$	homogeneous	1/120000/60000	CH_3CN, 50 °C, 12.4 h	95.2/4.8	20.9/10.5	12500	1010	Radical	7
$Os^{VI}(N)(quin)_2Cl$	homogeneous	1/100/2000	CH_2Cl_2/CH_3CO_2H, 23 °C, 0.42 h	93.6/6.4	2.3/47	47	113	Electrophilic	6
Fe-NHC	homogeneous	1/1000/100	CH_3CN, 25 °C, 1 h	87/13	11.2/1.1	11.2	11.2	Electrophilic	9
TS-1B	heterogeneous	1/114/1140	Sulfolane, 100 °C	94/6	8.3/83	95	/	Radical	16
PMO	heterogeneous	1/600/200	CH_3CN, 50 °C, 8 h	100/0	27.4/9.1	54.8	6.8	Radical	17
Oxo vanadium Schiff bases	heterogeneous	1/9523/4761	CH_3CN, 50 °C, 8 h	100/0	30.8/15.4	1466	183	Radical	18
$[Cu^{II}(tmpa)]^{2+}$@Al-MCM-41	heterogeneous	1/2100000/2100000	Acetone, 25 °C, 118 h	99/1	0.2/0.2	4320	36	Radical	19
Cu–Ag/C	heterogeneous	1/90/5	CH_3CN, 50 °C, 2 h	96/4	34.9/1.9	1.7	0.85	Radical	20
VPO@GO	heterogeneous	1/1200/400	CH_3CN, 60 °C, 8 h	100/0	32.8/10.9	131	16.4	Radical	21
$CuCr_2O_4$	heterogeneous	1/185/37	CH_3CN, 80 °C, 10 h	95/5	65/13	24	3	Radical	22
TS-1	heterogeneous	1/22/7.7	Acetone, 62 °C, 2 h	95/5	31/10.3	2.4	1.2	Radical	23
VSZ-5	heterogeneous	1/63/63	CH_3CN, 80 °C, 30 s	99/1	30.8/30.8	19.3	2315	Non-radical	**This**
VSZ-1	heterogeneous	1/634/634	CH_3CN, 80 °C, 30 s	77.2/22.8	7.5/7.5	47.6	5707	Non-radical	**work**

†C/O/S=molar ratio of catalyst/oxidant/substrate. ‡Ring/chain = mole ratio of ring-oxidized/side-chain oxidized products × 100%. §Ys (%)=[products (mol) /initial substrate (mol)]×100; Y_O(%)=[products (mol) /initial H_2O_2 (mol)]×100, also denoting the efficiency of H_2O_2.

Supplementary Table 7. Direct hydroxylation of toluene with H_2O_2 catalyzed by different catalysts.

Catalyst	Catalytic type	C/O/S†	Reaction conditions	R/C‡	Ys/Yo§(%)	o/m/pǀ	TON	TOF (h^{-1})	Mechanism	Ref.
$TBA_4[\gamma\text{-}HPW_{10}V_2O_{40}]$	homogeneous	1/40/ 2000	CH_3CN/tBuOH, 60 °C, 1 h	86/14	1/60	7/16/77	20	20	Electrophilic	10
$[Ni^{II}(tepa)]^{2+}$	homogeneous	1/50000/10000	CH_3CN, 60 °C,24 h	90/10	1/0.2	57/18/25	96	4	Radical	8
$[Cu_2(\mu\text{-}OH)(6\text{-}hpa)]^{3+}$	homogeneous	1/120000/30000	CH_3CN, 50 °C, 11.4 h	72/28	14.4/3.6	57/0/43	4320	380	Radical	7
$Os^{VI}(N)(quin)_2Cl$	homogeneous	1/100/2000	CH_2Cl_2/CH_3CO_2H, 23 °C, 0.42 h	99.8/0.2	3.7/74	57/7/36	74	178	Electrophilic	6
Fe-NHC	homogeneous	1/100/100	CH_3CN, 25 °C, 1 h	77.8/22.2	11.8/11.8	55/10/35	11.8	11.8	Electrophilic	9
$PV_2\text{-}SiW_{12}/Fe_2O_3$	heterogeneous	1/50/2500	EtOAc/t-BuOH, 60 °C, 4 h	99/1	0.5/25	9/19/72	12.5	3.1	Electrophilic	24
TS-1	heterogeneous	1/22.4/112	H_2O, 80 °C, 8 h	>99/0	8.3/41.5	34/0/66	9.3	1.2	Radical	25
VSZ-5	heterogeneous	1/63/63	CH_3CN, 80 °C,30 s	91.8/8.2	26.2/26.2	51:0:49	16.4	1969	Non-radical	**This work**

†C/O/S=molar ratio of catalyst/oxidant/substrate. ‡Ring/chain = mole ratio of ring-oxidized/side-chain oxidized products × 100%. §Y_s(%)=[products (mol) /initial substrate (mol)]×100; Y_O(%)=[products (mol) /initial H_2O_2 (mol)]×100, also denoting the efficiency of H_2O_2. ǀSelectivity=(mmol of a product/mmol of all products)×100.

Supplementary Discussion

Additional activity comparison:

Various homogeneous and heterogeneous catalysts have been developed for the benzene hydroxylation with H_2O_2. Supplementary Tables 6 and 7 list some of the most efficient ones. Compared with these previous works, VSZ-*n* catalyzed ring hydroxylation of arenes presented the following three unique features:

1) **Most rapid reaction rate**: The reaction was completed immediately after the addition of H_2O_2, rendering the most rapid catalytic system for the ring hydroxylation of arenes. The reaction time was shorter than 30 s, affording the highest TOF for the hydroxylation of benzene and its substituted derivations. Maximum TOF for the benzene hydroxylation was as high as 5707 h^{-1}, which not only greatly exceeds the ones over heterogeneous catalysts (1.2-183 h^{-1}, Supplementary Table 6) but also is larger than the most efficient homogeneous ones (3.5-1010 h^{-1}, Supplementary Tables 6). The TOFs in the hydroxylation of other arenes are also larger than previous ones (Supplementary Table 7)[6-10,24,25].

2) **High atom efficiency of the substrate and oxidant**: VSZ-*n* catalyzed ring hydroxylation of arenes offered high yield and selectivity under stoichiometric conditions, reaching high efficiency of both arenes and H_2O_2. By contrast, the reaction catalyzed by some effective homogeneous catalysts was performed by using high substrate/H_2O_2 ratio to inhibit over-oxidation of phenols, giving low yield based on substrate. Increasing the H_2O_2/substrate will decrease the selectivity. Excessive H_2O_2 was usually involved in the heterogeneous benzene hydroxylation, leading low efficiency for these catalytic systems. H_2O_2 efficiency was above 30% for VSZ-5 catalyzed benzene hydroxylation, higher than previous ones (Supplementary Tables 6 and 7).

2) **Well substrate compatibility**: Up to now, only several effective homogeneous catalysts were active in the hydroxylation of substituted benzenes. The POM catalyst $[\gamma\text{-}PW_{10}O_{38}V_2(\mu\text{-}OH)_2]^{3-}$ was active in the hydroxylation of various arenes with electron-donating groups but also effectively catalyzed the oxidation of phenol[22].

Though the activity in the benzene hydroxylation was unknown, it can be deduced that this catalyst cannot avoid the over-oxidation of phenol. The osmium(VI) nitrido Catalyst was highly selective for the alkylbenzenes but the selectivity for phenol in the benzene hydroxylation was 76.2%[6]. Further, benzene derivations with strong electron-withdrawing groups were hardly hydroxylated. Herein, hydroxylation of benzene, alkylbenzenes and halogenated aromatic hydrocarbons into the corresponding phenols was effectively catalyzed VSZ-5, giving satisfied yields and high selectivity. In short, VSZ-5 leads to the high efficiency for the chemo-selective ring hydroxylation of substituted benzenes and it also presents well substrates compatibility.

Supplementary References

1. Keller, D. E., Airaksinen, S. M. K., Krause, A. O., Weckhuysen, B. M. & Koningsberger, D. C. Atomic XAFS as a Tool To Probe the Reactivity of Metal Oxide Catalysts: Quantifying Metal Oxide Support Effects. *J. Am. Chem. Soc.* 129, 3189-3197 (2007).
2. Quadrelli, E. A. & Basset, J. M. On silsesquioxanes' accuracy as molecular models for silica-grafted complexes in heterogeneous catalysis. *Coordin. Chem. Rev.* 254, 707–728 (2010).
3. Kirillov, A. M. & Shul'pin, G. B. Pyrazinecarboxylic acid and analogs: Highly efficient co-catalysts in the metal-complex-catalyzed oxidation of organic compounds, *Coordin. Chem. Rev.* 257, 732–754 (2013).
4. Kirillova, M. V. et al. Mechanism of oxidations with H_2O_2 catalyzed by vanadate anion or oxovanadium(V) triethanolaminate (vanadatrane) in combination with pyrazine-2-carboxylic acid (PCA): Kinetic and DFT studies. *J. Catal.* 267, 140–157 (2009).
5. Alekar, N. A. et al. Kinetics and mechanism of selective hydroxylation of benzene catalysed by vanadium substituted heteropolymolybdates. *J. Mol. Catal. A: Chem.* 164, 181–189 (2000).
6. Lau, T. C. et al. Highly Selective and Efficient Ring Hydroxylation of Alkylbenzenes with H_2O_2 based on an Osmium (VI) Nitrido Catalyst. *Angew. Chem., Int. Ed.* 56, 12260–12263 (2017).
7. Tsuji, T. et al. Specific Enhancement of Catalytic Activity by a Dicopper Core: Selective Hydroxylation of Benzene to Phenol with Hydrogen Peroxide. *Angew. Chem. Int. Ed.* 56, 7779–7782 (2017).
8. Morimoto, Y., Bunno, S., Fujieda, N., Sugimoto, H. & Itoh, S. Direct hydroxylation of benzene to phenol using hydrogen peroxide catalyzed by nickel complexes supported by pyridylalkylamine ligands. *J. Am. Chem. Soc.* 137, 5867–5870 (2015).
9. Raba, A., Cokoja, M., Herrmann, W. A. & Kühn, F. E. Catalytic hydroxylation of benzene and toluene by an iron complex bearing a chelating di-pyridyl-di-NHC

ligand. *Chem. Commun.* 50, 11454–11457 (2014).

10. Kamata, K., Yamaura, T. & Mizuno, N. Chemo-and Regioselective Direct Hydroxylation of Arenes with Hydrogen Peroxide Catalyzed by a Divanadium - Substituted Phosphotungstate. *Angew. Chem., Int. Ed.* 51, 7275–7278 (2012).
11. Schwendt, P., Tatiersky, J., Krivosudsky, L. & Šimunekova, M. Peroxido complexes of vanadium. *Coordin. Chem. Rev.* 318, 135–157 (2016).
12. Ligtenbarg, A. G. J., Hage, R. & Feringa, B. L. Catalytic oxidations by vanadium complexes. *Coordin. Chem. Rev.* 237, 89–101 (2003).
13. Butler, A., Clague, M. J. & Meister, G. E. Vanadium Peroxide Complexes, *Chem. Rev.* 94, 625–638 (1994).
14. Mimoun, H. et al. Vanadium(V) Peroxo Complexes. New Versatile Biomimetic Reagents for Epoxidation of Olefins and Hydroxylation of Alkanes and Aromatic Hydrocarbons. *J. Am. Chem. Soc.* 105, 3101–3110 (1983).
15. Li, C. et al. The Dual Roles of Oxodiperoxovanadate Both as a Nucleophile and an Oxidant in the Green Oxidation of Benzyl Alcohols or Benzyl Halides to Aldehydes and Ketones. *Angew. Chem. Int. Ed.* 42, 5063–5066 (2003).
16. Balducci, L. et al. Direct oxidation of benzene to phenol with hydrogen peroxide over a modified titanium silicalite. *Angew. Chem., Int. Ed.* 42, 4937–4940 (2003).
17. Borah, P., Ma, X., Nguyen, K. T. & Zhao, Y. A vanadyl complex grafted to periodic mesoporous organosilica: A green catalyst for selective hydroxylation of benzene to phenol. *Angew. Chem. Int. Ed.* 51, 7756–7761 (2012).
18. Khatri, P. K., Singh, B., Jain, S. L., Sain, B. & Sinha, A. K. Cyclotriphosphazene grafted silica: a novel support for immobilizing the oxo-vanadium Schiff base moieties for hydroxylation of benzene. *Chem. Commun.* 47, 1610–1612 (2011).
19. Yamada, M., Karlin, K. D. & Fukuzumi, S. One-step selective hydroxylation of benzene to phenol with hydrogen peroxide catalysed by copper complexes incorporated into mesoporous silica–alumina. *Chem. Sci.* 7, 2856–2863 (2016).
20. Tian, K., Liu, W. J., Zhang, S. & Jiang, H. One-pot synthesis of a carbon supported bimetallic Cu–Ag NPs catalyst for robust catalytic hydroxylation of benzene to phenol by fast pyrolysis of biomass waste. *Green Chem.* 18,

5643–5650 (2016).

21. Borah, P., Datta, A., Nguyen, K. T. & Zhao, Y. $VOPO_4 \cdot 2H_2O$ encapsulated in graphene oxide as a heterogeneous catalyst for selective hydroxylation of benzene to phenol. *Green Chem.* 18, 397–401 (2016).
22. Acharyya, S. S., Ghosh, S. & Bal, R. Fabrication of three-dimensional (3D) raspberry-like copper chromite spinel catalyst in a facile hydrothermal route and its activity in selective hydroxylation of benzene to phenol. *ACS Appl. Mater. Interfaces* 6, 14451–14459 (2014).
23. Tanev, P. T., Chibwe, M. & Pinnavaia, T. J. Titanium-containing mesoporous molecular sieves for catalytic oxidation of aromatic compounds. *Nature* 368, 321–323 (1994).
24. Wang, Y. et al. Composites of $[\gamma\text{-}H_2PV_2W_{10}O_{40}]^{3-}$ and $[\alpha\text{-}SiW_{12}O_{40}]^{4-}$ supported on Fe_2O_3 as heterogeneous catalysts for selective oxidation with aqueous hydrogen peroxide. *Catal. Sci. Technol.* 5, 2602–2611 (2015).
25. Mukherjee, P., Bhaumik, A. & Kumar, R. Eco-friendly, Selective Hydroxylation of C-7 Aromatic Compounds Catalyzed by TS-1/H_2O_2 System under Solvent-free Solid-Liquid-Liquid-Type Triphase Conditions. *Ind. Eng. Chem. Res.* 46, 8657–8664 (2007).
